# Diversely evolved xibalbin variants from remipede venom inhibit potassium channels and activate PKA-II and Erk1/2 signaling

**DOI:** 10.1186/s12915-024-01955-5

**Published:** 2024-07-29

**Authors:** Ernesto Lopes Pinheiro-Junior, Ehsan Alirahimi, Steve Peigneur, Jörg Isensee, Susanne Schiffmann, Pelin Erkoc, Robert Fürst, Andreas Vilcinskas, Tobias Sennoner, Ivan Koludarov, Benjamin-Florian Hempel, Jan Tytgat, Tim Hucho, Björn M. von Reumont

**Affiliations:** 1 Toxicology and Pharmacology - Campus Gasthuisberg, University of Leuven (KU Leuven), Herestraat 49, PO Box 922, 3000 Louvain, Belgium; 2 Department of Anesthesiology and Intensive Care Medicine, University Cologne, Translational Pain Research, University Hospital of Cologne, Cologne, Germany; 3 Fraunhofer Institute for Translational Medicine and Pharmacology ITMP, Theodor-Stern-Kai 7, 60596 Frankfurt Am Main, Germany; 4 Institute of Pharmaceutical Biology, Goethe University Frankfurt, Max-Von-Laue-Str. 9, 60438 Frankfurt, Germany; 5 LOEWE Center for Translational Biodiversity Genomics (LOEWE-TBG), Senckenberganlage 25, 60325 Frankfurt, Germany; 6 Department of Bioresources, Fraunhofer Institute for Molecular Biology and Applied Ecology (IME-BR), Ohlebergsweg 14, 35394 Giessen, Germany; 7 Department of Informatics, Bioinformatics and Computational Biology, i12, Technical University of Munich, Boltzmannstr. 3, 85748 Garching, Munich, Germany; 8 Freie Unveristät Berlin, Veterinary Centre for Resistance Research (TZR), Robert-Von-Ostertag Str. 8, 14163 Berlin, Germany; 9 Faculty of Biological Sciences, Institute of Cell Biology and Neuroscience, Goethe, Frankfurt, Max-Von-Laue-Str 13, 60438 Frankfurt, Germany

**Keywords:** Venomous crustaceans, Marine venoms, Xibalbanus, Xibalbin_1_, Xibalbin_13_, Xibalbin_2_, Electrophysiology, HCI screening, ICK, Knottin

## Abstract

**Background:**

The identification of novel toxins from overlooked and taxonomically exceptional species bears potential for various pharmacological applications. The remipede *Xibalbanus tulumensis*, an underwater cave-dwelling crustacean, is the only crustacean for which a venom system has been described. Its venom contains several xibalbin peptides that have an inhibitor cysteine knot (ICK) scaffold.

**Results:**

Our screenings revealed that all tested xibalbin variants particularly inhibit potassium channels. Xib_1_ and xib_13_ with their eight-cysteine domain similar to spider knottins also inhibit voltage-gated sodium channels. No activity was noted on calcium channels. Expanding the functional testing, we demonstrate that xib_1_ and xib_13_ increase PKA-II and Erk1/2 sensitization signaling in nociceptive neurons, which may initiate pain sensitization. Our phylogenetic analysis suggests that xib_13_ either originates from the common ancestor of pancrustaceans or earlier while xib_1_ is more restricted to remipedes. The ten-cysteine scaffolded xib_2_ emerged from xib_1_, a result that is supported by our phylogenetic and machine learning-based analyses.

**Conclusions:**

Our functional characterization of synthesized variants of xib_1_, xib_2_, and xib_13_ elucidates their potential as inhibitors of potassium channels in mammalian systems. The specific interaction of xib_2_ with Kv1.6 channels, which are relevant to treating variants of epilepsy, shows potential for further studies. At higher concentrations, xib_1_ and xib_13_ activate the kinases PKA-II and ERK1/2 in mammalian sensory neurons, suggesting pain sensitization and potential applications related to pain research and therapy. While tested insect channels suggest that all probably act as neurotoxins, the biological function of xib_1_, xib_2,_ and xib_13_ requires further elucidation. A novel finding on their evolutionary origin is the apparent emergence of *X. tulumensis*-specific xib_2_ from xib_1_. Our study is an important cornerstone for future studies to untangle the origin and function of these enigmatic proteins as important components of remipede but also other pancrustacean and arthropod venoms.

**Supplementary Information:**

The online version contains supplementary material available at 10.1186/s12915-024-01955-5.

## Background

Venomous animals inject their toxic compounds into other organisms primarily for self-defense or predation [1, 2]. Numerous venoms comprise proteins that have evolved to modulate a range of physiological functions in their target organisms. Investigating these bioactivities may lead to pharmacological or agrochemical applications [1–5]. The majority of venoms and venom proteins that have been thoroughly studied mainly originate from iconic and terrestrial groups such as snakes, spiders, scorpions, and insects [2, 6–8]. Marine species have received limited research attention, with only a small number of fish and invertebrate species (such as sea anemones, jellyfish, cone snails, cephalopods, polychaetes, and recently nemerteans [6, 9–19]) being better studied. As venoms and their toxic proteins have independently evolved in various animal lineages, researching new lineages presents on the one hand an opportunity to identify novel venom compounds with interesting bioactivity and on the other hand to enhance our understanding of the evolution of convergent functional traits generally [2, 6, 20–25].

Only one venomous species of marine crustaceans has been described so far in more detail [26, 27]. *Xibalbanus tulumensis* belongs to the crustacean class Remipedia, which was first described over 40 years ago (Yager 1981) and currently comprises 28 extant species [28, 29]. However, the internal relationships of remipedes remain challenging [6, 26–28]. Phylogenomic analyses show that remipedes share a common ancestor with hexapods, making them a key taxon for comprehending insect evolution [30–34]. The biology and ecology of remipedes are not yet comprehensively understood, likely due to the extraordinary and secluded environment they inhabit as stygobionts in the marine saltwater regions of anchialine underwater cave systems [35].

**Fig. 1:**
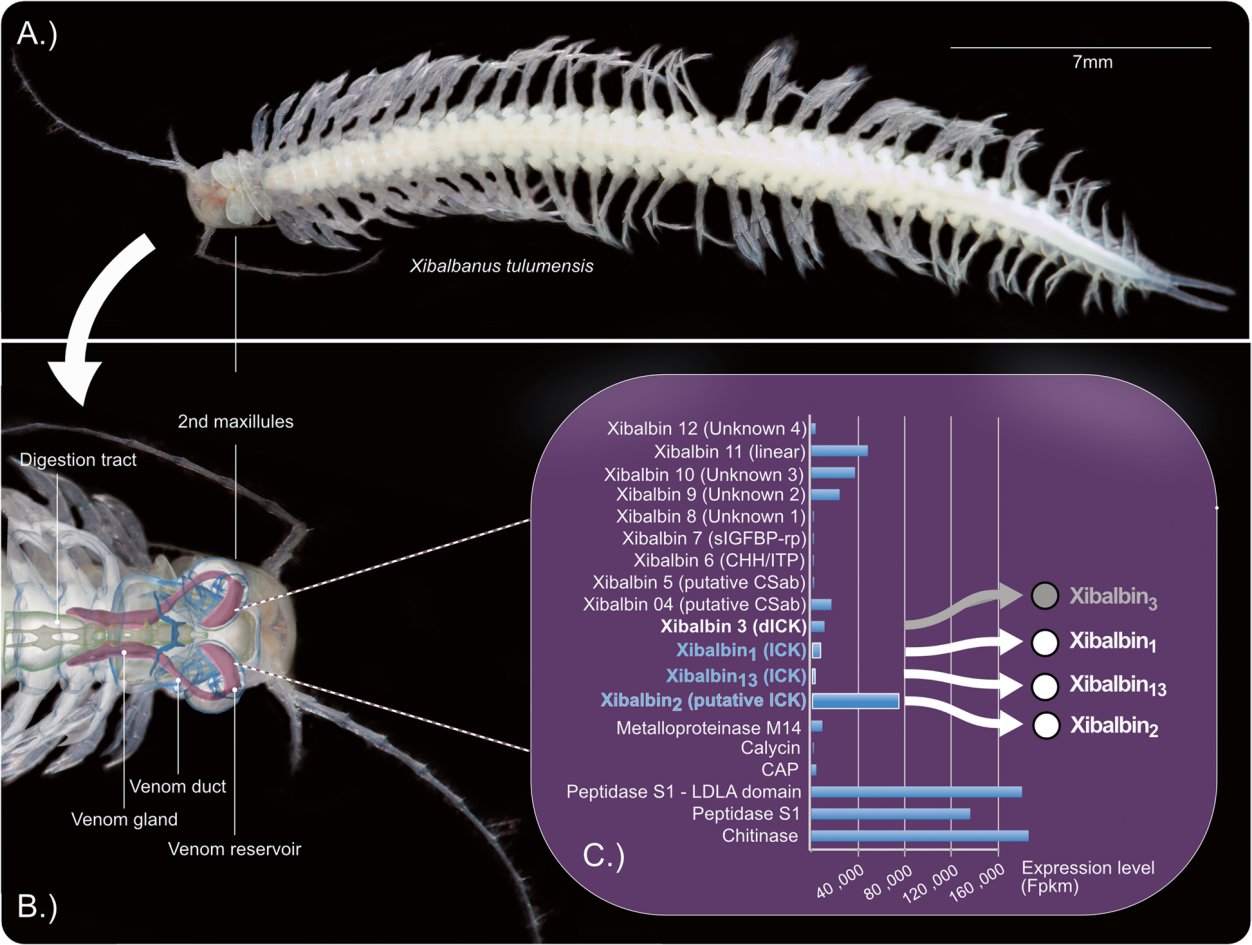
Overview of published data used for our study to investigate bioactivity of the higher expressed ICK-like peptides. **A** Habitus of an adult remipede *X. tulumensis*. **B** The anatomy of the venom system as described in von Reumont et al. [26] is illustrated by blending a synchrotron-based microcomputer tomography reconstruction into a light microscope picture. All components of the venom apparatus (venom gland, venom duct, and venom reservoir) are shown in magenta. **C** Proteo-transcriptome data used as a base for our study is illustrated in a modified graphic. Only proteome-verified transcripts of the venom profile described in von Reumont et al. [27] are shown with their expression levels on the *x*-axis. The three single ICK domain protein families xibalbin_1,_ xibalbin_2,_ and xibalbin_13_ are highlighted. The fourth family of knottin-like proteins, xibalbin_3_, which is a double ICK-like domain peptide and not the subject of this study, is indicated in gray

The venom system of *X. tulumensis* and its anatomy has been studied using synchrotron-based µ-computer tomography in the first comprehensive publication about remipede venom [26]; see Fig. 1.

Remipedes have large thoracic glands connected to reservoirs in their second maxillules, which release venom through an apical pore under a terminal claw [36]. This venom injection is facilitated by complex muscle arrangements [26, 36], though it is worth noting that direct observations of remipedes catching prey are lacking. Transcriptomics identified putative venom components, later detailed in the first proteo-transcriptomics study using squeezed-out gland lumen proteome [27]; see Fig. 1. Three main groups of peptides and proteins were found: enzymes, including chitinase, peptidase S1, and LDLa-domain containing peptidase S1; moderately expressed non-enzymatic proteins; and novel peptides (xibalbins). Many of these peptides resemble inhibitor cysteine knot peptides (ICKs or knottins), known for their robustness against enzymes, heat, and pH due to their characteristic cysteine scaffold that results in specific numbers of disulfide bridges [37, 38]. In various animal venoms, ICK peptides function as neurotoxins, hemolytics, or antibacterials, and they are also explored for pharmacological and agrochemical applications [3, 39].

The hypothesized mechanism of envenomation by remipedes, based on proteo-transcriptome data sequence similarities [27], suggests that xibalbin1 (xib_1_), xibalbin2 (xib_2_), and xibalbin13 (xib_13_) ICK-like protein families, and the double-ICK-like xibalbin3 (xib_3_), could act as putative neurotoxins that rapidly cause paralysis in prey during an attack. Simultaneously, the proteins and enzymes break down internal tissues and structures, resulting in prey liquefaction and subsequent feeding by remipedes [27]. However, it should be noted that bioactivity tests for the venom compounds of remipedes, particularly the ICK-like peptides, have been lacking so far.

In this study, we investigate the bioactivity of synthetic variants of xib_1_, xib_13_, and xib_2_ focused on possible application potential, examining their cytotoxicity (including cancer cell lines), ability to modulate ion channels, and impact on sensory neurons. Our research explores also insight into their biological functions by testing insect targets. Furthermore, we shed first light on the diversity and origin of ICK-like peptides in remipedes by including sequences from further species besides *X. tulumensis* using phylogenetic and machine learning approaches.

## Results

### Xib_1_ is unique in remipedes while xib_13_ is similar to other ICKs, whereas xib_2_ and xib_3_ are specific to *Xibalbanus*

**Fig. 2:**
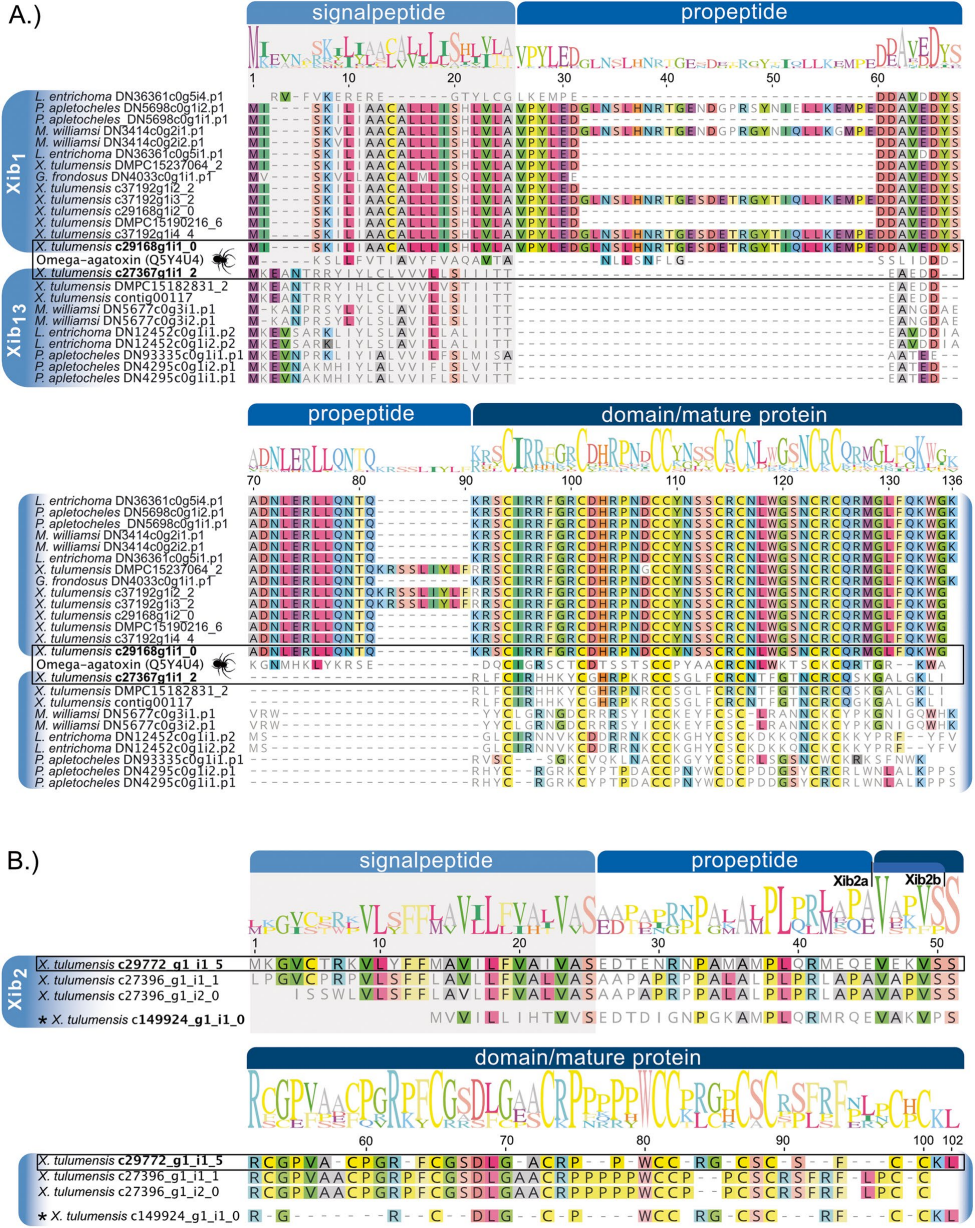
Alignments of xibalbin variants. **A** Xib_1_ and xib_13_ sequences identified in all remipede transcriptomes and a representative omegatoxin sequence from funnel web spiders (*Agelena*). Signal peptide, propeptide, and mature sequence are indicated on top of the sequences. The proteome-verified xib_1_ and xib_13_ sequences that were synthesized from *X. tulumensis* are highlighted by black boxes and shown in bold. **B** Xib_2_ sequences identified in *X. tulumensis*. The sequence that was proteomically verified and synthesized is indicated by a black box. We synthesized two variants that differ in the prediction of the mature sequence as shown. Signal peptide, propeptide, and mature regions in **A** and **B** were separately aligned using mafft-L-INS-I. The asterisk indicates the intermediate xibalbin form that is more similar to ten-cysteine scaffolded sequences while having only eight cysteines

To broaden the scope of our study beyond the single species *X.* *tulumensis*, we examined potential variants of xib_1_, xib_2,_ xib_13,_ and xib_3_ (double ICK, not tested in this study) in four other remipedes. This analysis was based on de novo assembled transcripts from *X.* *tulumensis* from which secreted proteins were identified proteomically [27]; see Figs. 1 and 2. Additionally, we used transcriptomes of whole animals, including venom systems, from *Lasionectes entrichoma*, *Morlockia williamsi*, *Godzillignomus frondosus*, and *Pleomothra apletocheles* that have been published [33, 40]. To identify venom proteins in these published data, an automated search pipeline [41] was employed utilizing hmmer-based identification resulting in final alignments of xibalbins (Fig. 2); see Methods for further details.

Transcripts of xib_1_ were found in four of the five remipede transcriptomes, exhibiting higher sequence variation than xib_13_. Both share an identical scaffold with eight cysteines in the mature peptide (see Fig. 2). Similar sequences to xib_2_ or xib_3_ (which feature a double ICK-like domain) were not recovered in the four other remipedes, except for *X.* *tulumensis* (see Fig. 2). No other ICK groups with different scaffolds were found, such as the known six-cysteine backbones from insects or cone snails [26, 27].

### Prediction and synthesis of xib_1_, xib_2_, and xib_13_ mature sequences

The mature sequences for chemical synthesis were predicted using the xibalbin alignments including all transcripts from the five available remipede species; see Fig. 2. Only transcripts with signal peptides described in the proteo-transcriptomic study [27] were considered. The transcripts obtained from the four reassembled novel remipede transcriptomes (besides *X. tulumensis)* guided us additionally to identify mature sequences of representative peptide variants; see Fig. 2 and “Methods.” The xibalbin_2_ variants that we name xib_2a_ and xib_2b_ derive from the same sequence but differ in two ambiguously predicted propeptide cleavage sites (Fig. 2). All xibalbin variants were produced synthetically by non-selective refolding; for details, see “Methods.” It has to be noted that the purity of the products differs (Xib_1_: 88.0%, xib_12_: 84.9% purity, xib_2a_: 99.9%, xib_2b_: 74.4%) and that the purity < 75% for xib_2b_ is less ideal for bioactivity tests; see Additional File 1: Figure S1.

### Xib_1_, xib_2_, and xib_13_ strongly inhibit voltage-gated potassium channels and mildly inhibit selected sodium channels while they show no inhibition of calcium channels

**Fig. 3:**
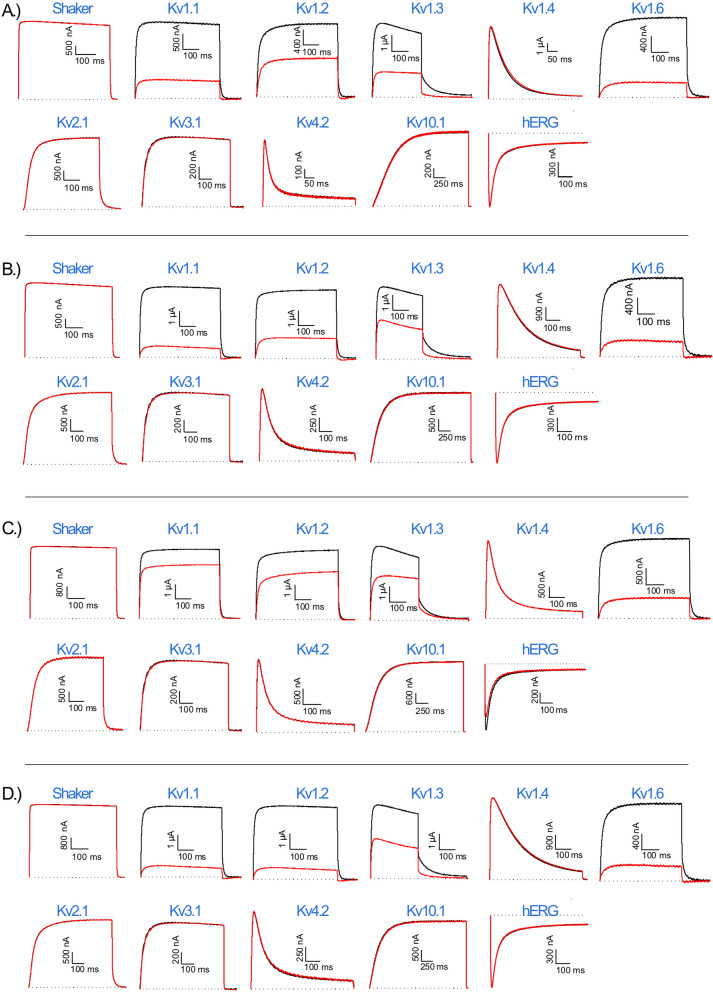
Electrophysiological characterization of xib_1_ (**A**), xib_13_ (**B**), xib_2a_ (**C**), and xib_2b_ (**D**), at 1 µM, on a panel of Kv channels. The black lines represent the control condition, while the red lines indicate the current obtained after the addition of each peptide. The dotted lines represent the 0 current level. The graphs illustrate the effects obtained in a series of at least three independent experiments (*n* ≥ 3); see “Methods” and Additional File 2: Table S1 for individual data values

**Fig. 4:**
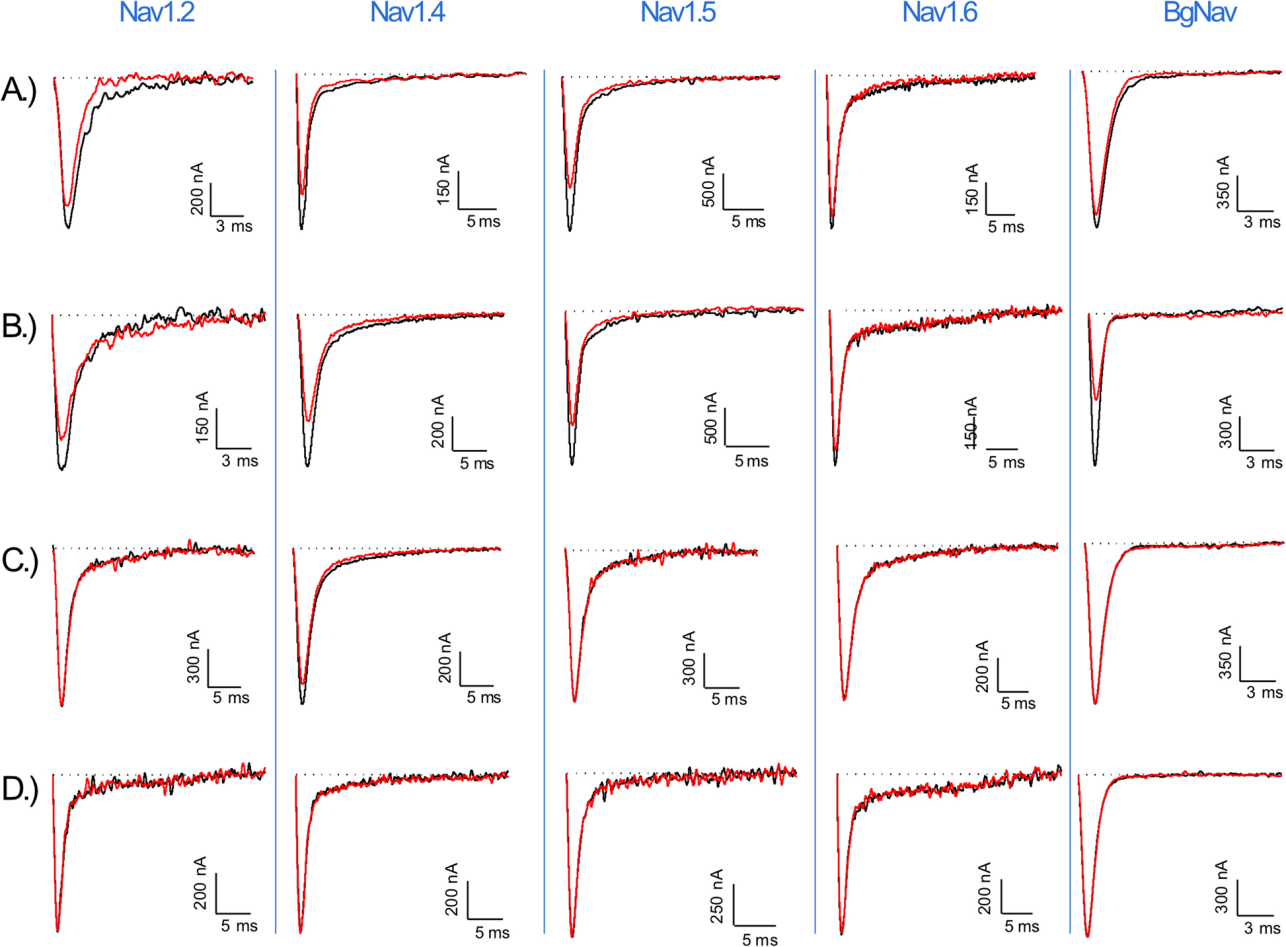
Electrophysiological characterization of xib_1_ (**A**), xib_13_ (**B**), xib_2a_ (**C**), and xib_2b_ (**D**), at 1 µM, on a panel of Nav channels. The black lines represent the control condition, while the red lines indicate the current obtained after the addition of each peptide. The dotted lines represent the 0 current level. The graphs illustrate the effects obtained in a series of at least three independent experiments (*n* ≥ 3); see “Methods” and Additional File 2: Table S1 for individual data values

**Fig. 5:**
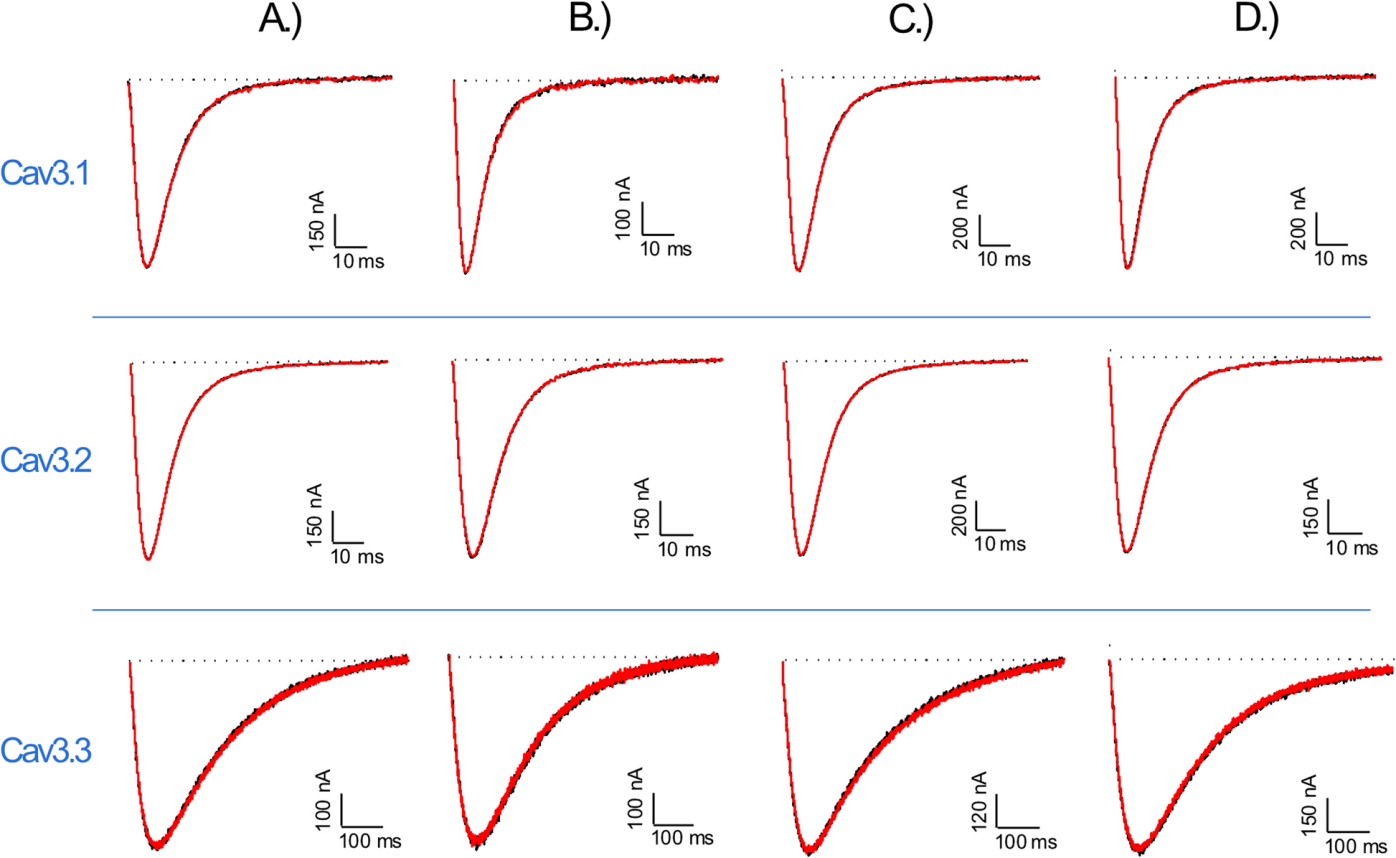
Electrophysiological characterization of xib_1_ (**A**), xib_13,_ (**B**), xib_2a_ (**C**), and xib_2b_ (**D**), at 1 µM, on Cav3.1, Cav3.2, and Cav3.3. The black lines represent the control condition, while the red lines indicate the current obtained after the addition of each peptide. The dotted lines represent the 0 current level. The graphs illustrate the effects obtained in a series of at least three independent experiments (*n* ≥ 3); see “Methods” for details

We tested the xibalbin variants on a broad selection of voltage-gated potassium (Fig. 3), sodium (Fig. 4), and calcium channels (Fig. 5). The relevant channels were exogenously expressed in *Xenopus laevis* oocytes, and their activities were measured using the two-electrode voltage clamp technique.

**Fig. 6:**
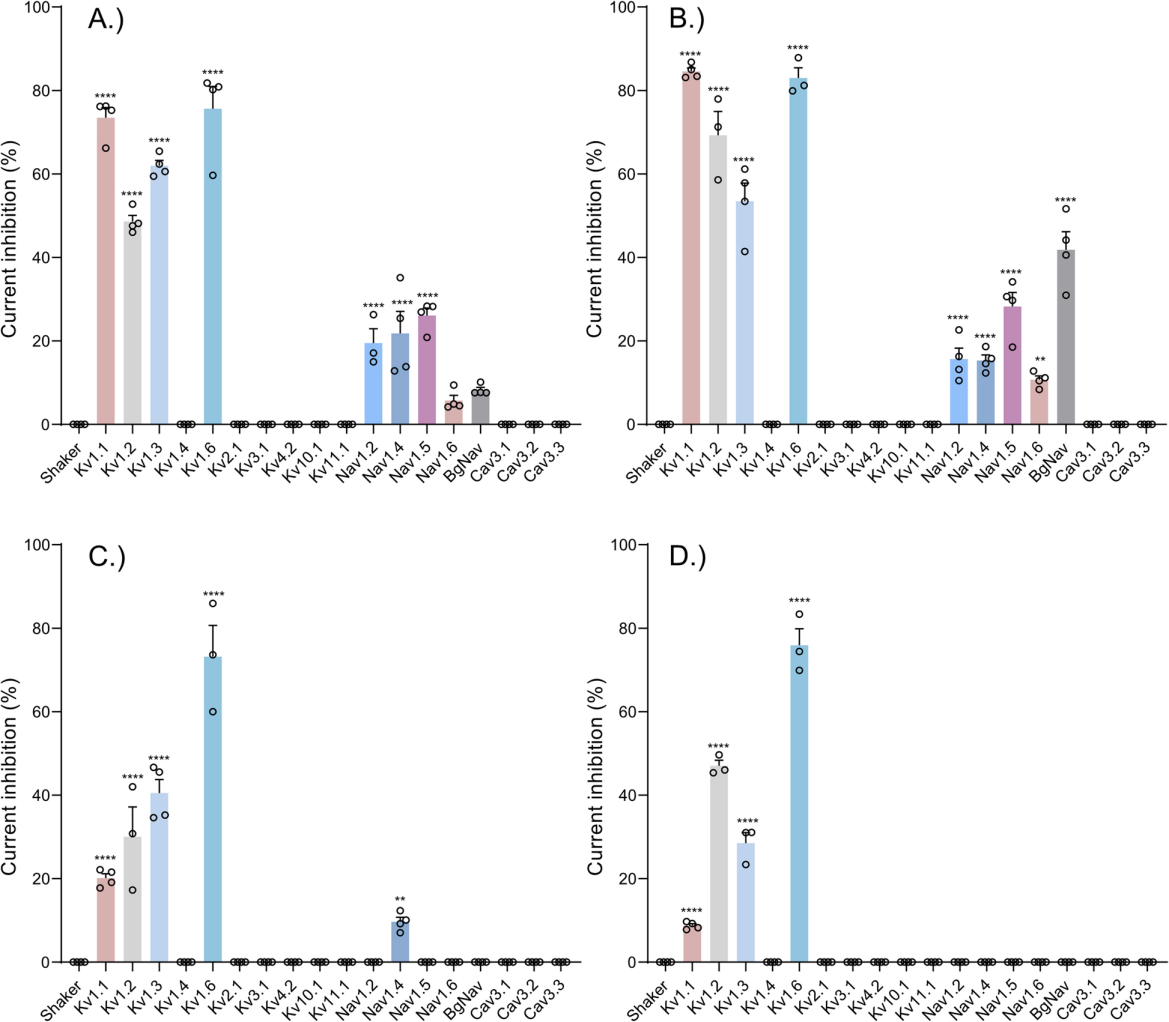
Effect of xib_1_ (**A**), xib_13_ (**B**), xib_2a_ (**C**), and xib2_b_ (**D**), at 1 µM versus control, on a panel of voltage-gated potassium (Kv), sodium (Nav), and calcium (Cav) channels. (*n* ≥ 3) ± S.E.M.; S.E.M standard error of the mean. **p < 0.01; ****p < 0.0001. Differences in ionic currents between control and sample conditions were compared by one-way ANOVA, followed by Dunnet’s multiple comparisons test. Differences were considered statistically significant when *p* < 0.01. See “Methods” and Additional File 2: Table S1 for individual data values

Xib_1_ and xib_13_ were found to exhibit a potent inhibition on voltage-gated potassium channels (Kvs) and a mild inhibition on voltage-gated sodium channels (Nav). In contrast, xib_2a_ and xib_2b_ displayed a preference for a limited range of Kvs. Xib_2a_ specifically targeted the Nav1.4 isoform with a high degree of selectivity, while xib_2b_ inhibited solely Kvs. Notably, no detectable activity was observed on T-type calcium channels Cav3.1, Cav3.2, and Cav3.3 (Fig. 6).

Among the Kvs, the highest inhibitory activity of xib_1_ (1 µM) was seen on Kv1.6 (74.1 ± 4.1%), followed by Kv1.1 (72.8 ± 3.3%), Kv1.3 (62.0 ± 1.3%), and Kv1.2 (50.2 ± 2.6%). On Navs, Nav1.5 was the most affected isoform (26.1 ± 1.8%), while Nav1.4 (21.8 ± 3.3%), Nav1.2 (15.8 ± 4.4%), BgNav (8.2 ± 0.6%), and Nav1.6 (5.7 ± 1.2%) were also inhibited. Conversely, Kv1.1 was the most affected Kv isoform by xib_13_ (85.1 ± 1.7%), followed by Kv1.6 (83.0 ± 2.4%), Kv1.2 (69.3 ± 5.7%), and Kv1.3 (53.5 ± 4.4%). Regarding Nav isoforms, the insect channel BgNav was the most sensitive for this peptide (41.8 ± 4.3%), followed by Nav1.4 (33.0 ± 2.4%), Nav1.5 (28.2 ± 3.4%), Nav1.2 (15.6 ± 2.6%), and Nav1.6 (10.7 ± 0.9%).

Xib_2a_ and xib_2b_ demonstrated a predominant interaction with Kv channel isoforms, specifically Kv1.6, which exhibited the most notable effect (73.2 ± 7.5% and 75.9 ± 4.0%, for xib_2a_ and xib_2b_, respectively). Xib_1a_ also inhibited Kv1.3 (40.6 ± 6.0%), Kv1.2 (30.0 ± 7.1%), and Kv1.1 (20.0 ± 2.2%). Additionally, xib_2a_ displayed a minor degree of inhibition on Nav1.4 (9.7 ± 1.1%) in addition to the Kv channels. Xib_2b_ exclusively affected Kvs, while also inhibiting Kv1.2 (47,0 ± 1.6%), Kv1.3 (28.5 ± 2.5%), and Kv1.1 (8.7 ± 0.5%).

**Fig. 7:**
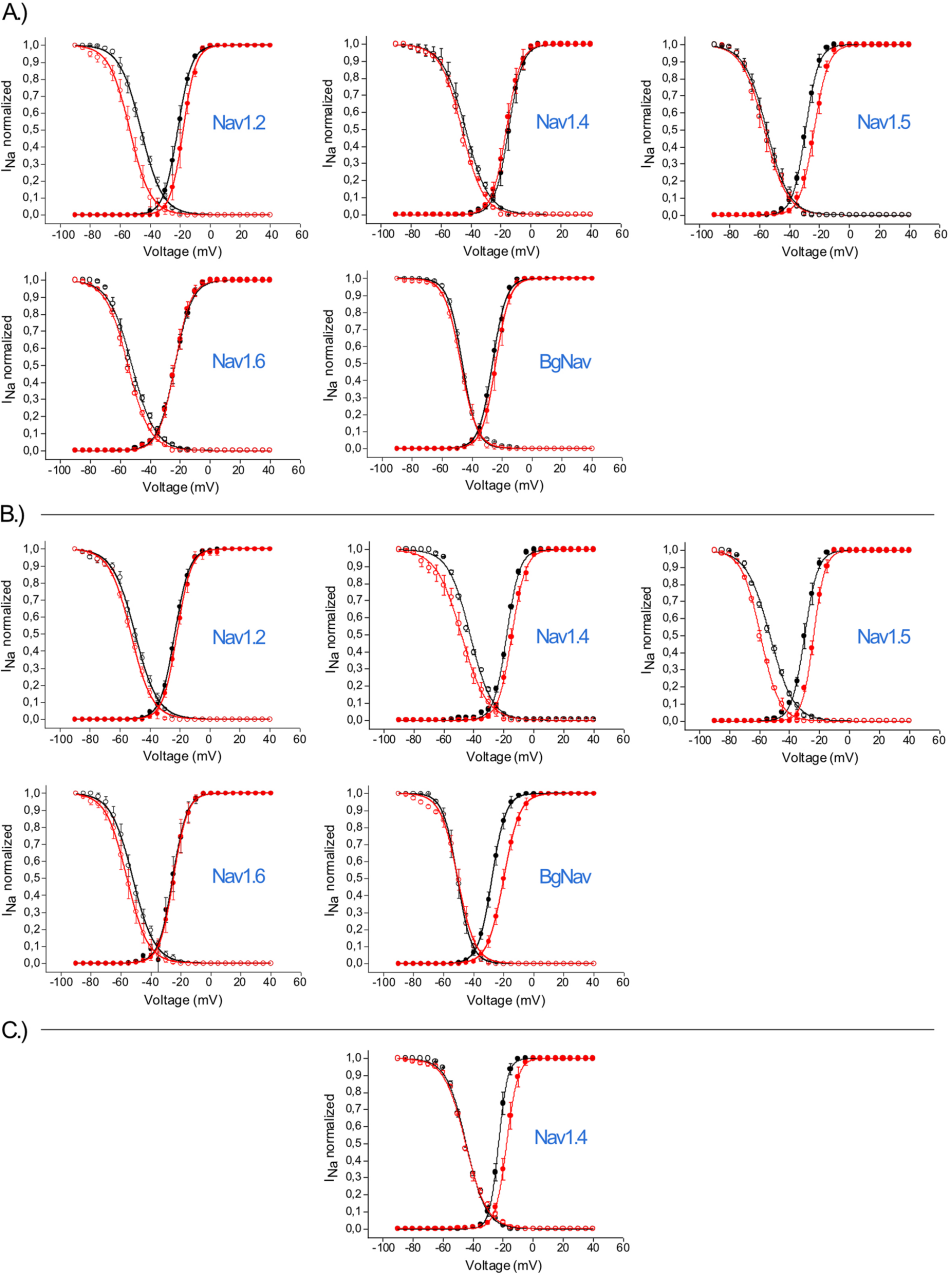
Activation (closed symbols) and steady-state inactivation (open symbols) curves in control (black) and toxin (red) condition for each Nav channel in the presence of 1 µM of xib_1_ (**A**), xib_13_ (**B**), and xib_2a_ (**C**). (*n* ≥ 3) ± SEM; SEM standard error of the mean. Statistics were calculated using one-way ANOVA, followed by Tukey’s multiple comparisons test. Differences were considered statistically significant when *p* < 0.01; see Tables 1 and 2. See “Methods” and Additional File 2: Table S1 for individual data values

Furthermore, the peptide’s impact on Nav channels was assessed by analyzing the current–voltage relationships of xib_1_, xib_13_, and xib_2a_, providing insights on its ion channel blocking mechanisms (Fig. 7). The data at *V*_half_ indicates that the peptides serve primarily as pore blockers for the majority of Nav isoforms since most blocked Nav channels did not present a notable shift in their activation and steady-state inactivation curves, when compared to control (Tables 1 and 2).

**Table 1:** Shifts in current–voltage relationships of voltage-gated sodium channels in control (ND96) and in the presence of 1 µM xib_1_. **p < 0.01, Fig. 7A

	***V***_**half**_** activation (mV ± SEM)**			***V***_**half**_** inactivation (mV ± SEM)**		
Channel isoform	Control (ND96)	Xib_1_ (1 µM)	Shift (mV)	Control (ND96)	Xib_1_ (1 µM)	Shift (mV)
**Nav1.2**	− 21.5 ± 0.1	− 19.3 ± 0.1	3.8	− 45.9 ± 0.2	− 55.5 ± 0.2	− 9.6^**^
**Nav1.4**	− 14.9 ± 0.1	− 16.4 ± 0.1	− 1.5	− 43.6 ± 0.2	− 46.0 ± 0.2	− 2.4
**Nav1.5**	− 29.3 ± 0.1	− 23.5 ± 0.1	5.8 (ns)	− 56.6 ± 0.2	− 55.1 ± 0.2	1.5
**Nav1.6**	− 25.5 ± 0.1	− 23.6 ± 0.1	1.9	− 52.2 ± 0.3	− 54.8 ± 0.1	− 2.6
**BgNav**	− 26.2 ± 0.1	− 24.1 ± 0.1	2.1	− 46.4 ± 0.1	− 47.4 ± 0.1	− 1.0

**Table 2:** Shifts in the current–voltage relationship of voltage-gated sodium channels in control (ND96) and in the presence of 1 µM xib_13_. **p < 0.01; ***p < 0.001, Fig. 7 B

	***V***_**half**_** activation (mV ± SEM)**			***V***_**half**_** inactivation (mV ± SEM)**		
Channel isoform	Control (ND96)	Xib_13_ (1 µM)	Shift (mV)	Control (ND96)	Xib_13_ (1 µM)	Shift (mV)
**Nav1.2**	− 23.3 ± 0.1	− 21.8 ± 0.1	1.5	− 49.7 ± 0.2	− 52.5 ± 0.2	− 2.8
**Nav1.4**	− 18.1 ± 0.1	− 14.7 ± 0.1	3.4	− 42.5 ± 0.2	− 48.4 ± 0.2	− 5.9**
**Nav1.5**	− 29.9 ± 0.1	− 24.0 ± 0.1	5.9***	− 53.1 ± 0.2	− 59.4 ± 0.1	− 6.3***
**Nav1.6**	− 25.3 ± 0.3	− 24.8 ± 0.1	0.5	− 52.0 ± 0.2	− 55.9 ± 0.1	− 3.9
**BgNav**	− 27.6 ± 0.1	− 19.9 ± 0.1	7.7***	− 50.6 ± 0.1	− 50.4 ± 0.2	0.2

However, xib_1_ significantly shifted the *V*_half_ of inactivation of Nav1.2 (− 45.9 ± 0.2 mV to − 55.5 ± 0.2 mV) and the *V*_half_ of activation of Nav1.5 (− 29.3 ± 0.1 mV to − 23.5 ± 0.1 mV). Additionally, Xib_13_ significantly shifted the *V*_half_ of inactivation of Nav1.4 (− 42.5 ± 0.2 mV to − 48.4 ± 0.2 mV) and Nav1.5 (− 53.1 ± 0.2 mV to − 59.4 ± 0.1 mV), as well as the *V*_half_ of activation of Nav1.5 (− 29.9 ± 0.1 mV to − 24.0 ± 0.1 mV) and the insect channel BgNav (− 27.6 ± 0.1 mV to − 19.9 ± 0.1 mV).

Xib_2a_ solely inhibited the Nav1.4 isoform (Fig. 7 C) and significantly altered its *V*_half_ of activation (− 23.0 ± 0.1 mV to − 17.6 ± 0.1 mV), with no notable shift of the *V*_half_ of inactivation (− 44.6 ± 0.2 mV to − 44.9 ± 0.3 mV). Notably, these data suggest that besides interacting with the pore region of these Nav isoforms, by reducing the flow of ions through the channel, xibalbins may also interact with the voltage sensor domain of some Nav isoforms (Figs. 6 and 7). Nonetheless, additional studies are needed to fully elucidate their precise mechanism of action.

### Xib_1_, xib_13_, and xib_2_ do not show overt cytolytic or cytotoxic activity

Having characterized the primary structure of xibalbins and having tested them on selected ion channels, we aimed to determine the biological activity of xibalbins on the primary cells of adult male rats. The diverse interactions with voltage-gated ion channels suggest that xibalbins have effects not only on electrically active cells such as neurons and cardiomyocytes but also on kidney cells. Consistent with previous studies on other ICK toxins, we evaluated the activity of xibalbins on cultured sensory neurons [42–44].

We initially determined the cytotoxic effects of xibalbins on primary sensory neurons from adult male rats cultured in vitro [2]. Sensory neurons exhibit increased sensitivity to changes in size or loss of attachment following exposure to cytotoxic levels of calcium [45]. We exposed overnight cultures of dissociated rat dorsal root ganglions (DRGs) to xibalbins for 5 and 30 min. Subsequently, all cells were fixed and immunocytochemically stained for the neuronal marker UCHL1 to identify neurons. The entire culture was digitally scanned using high content imaging (HCI) microscopy, and cell numbers, UCHL1 staining per cell, and cell size was analyzed. No loss of neurons was induced by either of the tested xibalbins, even at the highest concentration of 0.8 µg/µL (Additional File 3: Figure S2). Additionally, no differences were observed in the size distribution (Additional File 4: Figure S3 A, C) or the staining of the neuronal marker UCHL1 (Additional File 4: Figure S3 B, D). We further investigated the potential toxicity of xibalbins in monocyte/macrophage-like cells (RAW264.7) and human microvascular endothelial cells (HMEC); see Additional File 5: Figure S4 The viability of RAW264.7 cells was detected using a formazan-based assay, while the proliferation of HMECs was assessed by measuring the number of crystal violet-stained cells. The tests did not detect any significant cytotoxic activity of the tested xibalbins.

### Xib_1_ and xib_13_ activate PKA-II and Erk1/2 in sensory neurons, xib_2_ does not

**Fig. 8:**
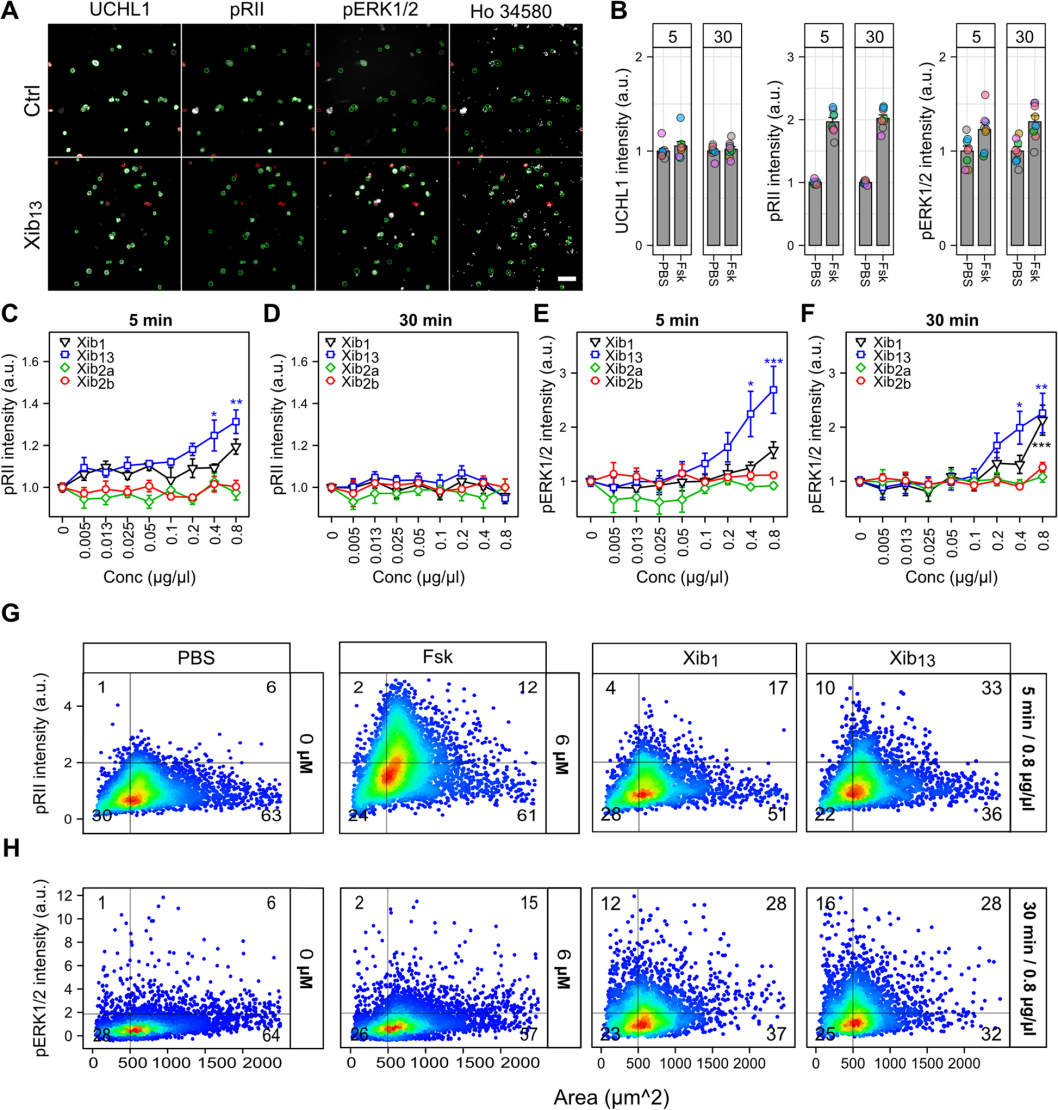
**A** Representative images of rat DRG neurons stimulated with PBS (Ctrl) and xib_1b_ (0.8 µg/µL). Cells were stained for UCHL1, pRII, pERK1/2, and Hoechst followed by fixation and staining with standard immunocytochemistry protocols. Green-encircled neurons indicate automatically selected objects. Scale bar: 100 µm. **B** Mean intensities of UCHL1, pRII, and pERK1/2. The same UCHL1 intensities were observed among tested groups. Forskolin (Fsk), an activator of adenylyl cyclase, was used as a positive control. Fsk at 6 µM concentration induced an increase in pRII and pERK1/2 intensities at 5 and 30 min. Number of analyzed cells per replica: 750 ± 200. Eight independent replicas. **C–F** Concentration responses of xibalbin toxins (0.005 to 0.8 µg/µL) and mean intensities of pRII and pERK1/2 at 5 min and 30 min. **G–H** Size versus pRII and pErk1/2 intensities of cells treated with 0.8 µg/µL of xib_1_ and xib_13_. Small-sized cells were responding to xibalbins. Statistical significance was assessed using one-way ANOVA with Dunnett’s post hoc test. Data represent mean ± S.E.M (standard error of the mean), *n* = 4, see “Methods” for details and Additional File 6: Table S2 for individual data values

Knottins have been demonstrated to modify neuronal activity by acting on voltage-gated ion channels. To gauge sensory neuron activity in response to various activating stimuli, including electrical activity, phosphorylation state detection of protein kinase A type II (PKA-II) and MAP kinase Erk1/2 can act as surrogate measurements [42, 46–48]. Consequently, we tested for increased phosphorylation states in a concentration-dependent manner following exposure to xibalbins. Dissociated DRG neurons were cultured overnight and then exposed to increasing concentrations of the corresponding xibalbins for 5 min and 30 min, respectively. As a positive control, forskolin (Fsk) was also used to induce cAMP synthesis [49, 50]. The activity of PKA-II was monitored by antibodies directed against the phosphorylation site of the inhibitory regulatory subunits RIIα/β, which is exclusively accessible when the catalytic kinase domain is released during kinase activation [42]. For measuring Erk1/2 activity, phospho-sites on Erk1/2 (T202/Y204) were monitored through immunofluorescence, as these sites are phosphorylated during activation [51, 52]. Cellular images were captured using HCI microscopy (Fig. 8A), and the average intensity of each phospho-antibody was quantified (Fig. 8B).

Xib_13_ induced an increase in phospho-PKA-II intensity in a concentration-dependent manner, following exposure for 5 min at 0.4 µg/µL, up to 1.21-fold (*q* = 3.29). Similarly, at a concentration of 0.8 µg/µL (148 and 74 µM), the intensity increased up to 1.21-fold (*q* = 4.17); see Fig. 8C. No change was observed after 30 min of exposure (Fig. 8D). The phosphorylation signal of Erk1/2 (pErk1/2) increased significantly with xib13 after 5 and 30 min, up to 2.5- and 2.3-fold, respectively (*q* = 4.54 and 4.32). This effect occurred at a concentration of 0.8 µg/µL (148 µM); see Fig. 8E, F.

At the highest tested concentration of 0.8 µg/µL(154 µM), xib_1_ caused a significant increase in pErk1/2 intensity by 2.17-fold (*q* = 4.07); see Fig. 8F. However, no changes were observed in the intensity of either phospho-PKA-II or pErk1/2 with xib2a and xib2b exposure at either of the two exposure times (Fig. 8C-G).

### Xibalbins induce Erk1/2 and PKA-II activation in primary nociceptive and non-nociceptive sensory neurons

Cultures of primary sensory neurons consist of a variety of different neuron subtypes. This includes large-sized non-nociceptive and small-sized nociceptive neurons. To characterize if xibalbins activate either one or both neuron subtypes, we analyzed the response according to the cell size. Forskolin used as a positive control to increase phospho-PKA-II activity did not show a prevalence and activated both small and large-sized sensory neurons (Fig. 8G, H). Upon testing with the highest concentrations, xib_1_ and xib_13_ also showed increased phosphorylation signals in both small-sized and large-sized sensory neurons (Fig. 8, H). Therefore, it can be concluded that xib_1_ and xib_13_ act on both nociceptive and non-nociceptive neurons.

### No signaling induced by pain-inducing mediators

Xibalbins that modulate sensitization signaling in non-nociceptive and nociceptive sensory neurons suggest the involvement of these toxins in sensation. Therefore, we conducted further tests on pain-related cellular activity including also non-neuronal cells such as HEK293, RAW264.7 macrophage cell line, and leukocytes. Pain-inducing mediators such as bradykinin, prostaglandin E2, LPS, and TNF act by, e.g., increasing Ca^2+^ influx through calcium channels, leading to a significant increase in intracellular calcium ([Ca^2+^]_i_), by an increase of cyclic adenosine monophosphate (cAMP) synthesis, and/or by induction of NO synthesis [53]. Such elevated levels of intracellular Ca^2+^, cAMP, or NO can contribute directly and indirectly to an increase in neural activity. Consequently, this can lead to a heightened perception of pain, which is relayed to the central nervous system via direct and indirect pathways.

We examined the potential of xibalbins in a concentration range of 0.25 to 25 µg/ml regarding their effects on Ca^2+^ influx in HEK293 cells, which express calcium channels. Forskolin, utilized as the positive control, increased [Ca^2+^]_i_ in HEK293 cells [54]. However, exposure to none of the four xibalbin variants by itself did increase [Ca^2+^]_i_ as measured by Fluo-8 calcium imaging assay. Additionally, none of the xibalbin variants modulated forskolin-induced [Ca^2+^]_i_ increase (Additional File 7: Figure S5 A). We also evaluated the effect of xibalbins on cAMP synthesis. However, xibalbins had no effect on increasing cAMP synthesis in HEK293 cells or on preventing the forskolin-induced cAMP synthesis (Additional File 7: Figure S5 B).

Nitric oxide has a complex and diverse role in pain modulation [55]. Our study reveals that xibalbins did not induce NO synthesis in RAW264.7 macrophages and is ineffective in preventing LPS-induced NO synthesis. Additionally, xibalbins showed no cytotoxic effects in RAW264.7 macrophages (Additional File 7: Figure S5 C). Finally, we analyzed the effects of xibalbins on the leukocyte adhesion to the vascular endothelium, which is a critical step in the inflammatory response of inflamed tissues. Xibalbins were analyzed for their ability to alter the adhesion of human monocytic (THP-1) cells onto a TNF-activated endothelial cell monolayer (Additional File 5: Figure S4). Thus, we do not find xibalbins to have an impact on Ca^2+^, cAMP, and NO signaling in the cell types analyzed. They do not interfere with the adhesion of leukocytes to endothelial cells. All individual values for these experiments are given in Additional File 8: Table S3.

### Diversity and evolutionary origins of ICK-like xibalbins

To explore the diversity and evolutionary origins of all ICK-like xibalbins, we aligned all full protein sequences from the five remipede species (see Fig. 2) with published ICK peptide sequences from arthropods, see “Methods”. These complementary arthropod sequences include confirmed and predicted ICK peptides from venomous and non-venomous crustaceans, insects, myriapods, and chelicerates. The additional sequences were acquired from two studies that collated ICK peptides from pancrustaceans and arthropods [27, 56] to reconstruct a maximum likelihood-based phylogenetic tree; see “Methods”. Although our topology remains unresolved in some, especially deeper nodes, we can infer important insights related to the evolution of xibalbins.

**Fig. 9:**
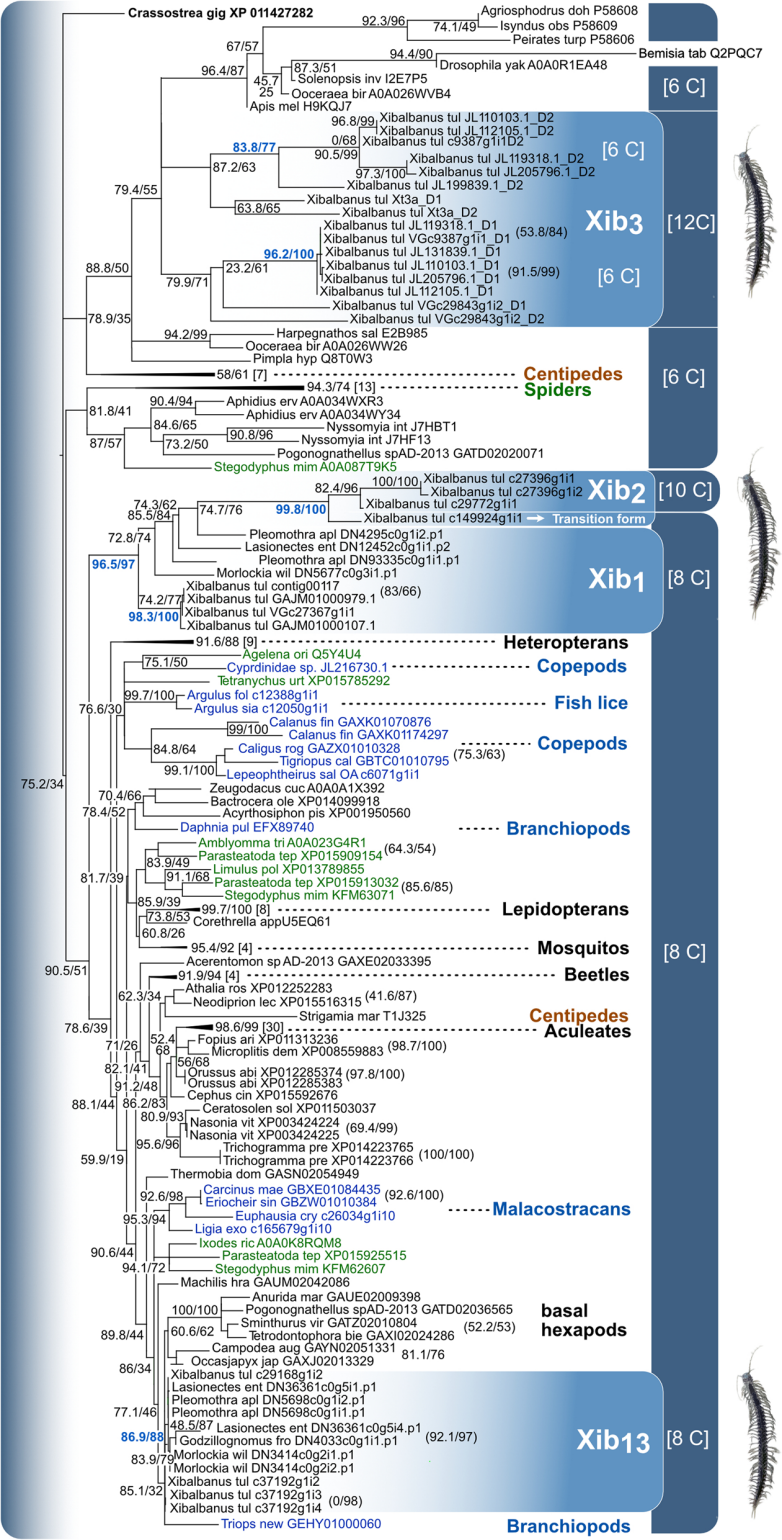
Phylogeny of ICK-like proteins in major arthropod groups and knottin-like xibalbins in remipedes. Nodes for which both support values are below 50 are shown in multifurcation, the first number gives the SH-aLRT support in percent, and the second number the fast bootstrap support. The relevant node values for xibalbin protein clades are printed in bold blue. Chelicerates are green, myriapods brown, and crustaceans in blue, while hexapods are colored in black. Major groups are indicated. The cysteine scaffold of ICK-like sequences is shown in white in the dark blue bars. The tree was calculated in IQTREE (-m MFP -alrt 25,000 -B 25000 -bnni -T Auto); for further details, see “Methods”. We summarized several nodes for better visualization (triangles); see the full tree file (Additional File 9: Figure S6)

Our findings demonstrate that xib_13_ sequences, which are present in all five remipedes, are located in a well-supported clade (88 fast bootstraps) indicating a remipede-specific family that clusters with sequences from the notostracan crustacean *Triops* and basal hexapods that display a similar eight-cysteine scaffold akin to xib_13_ (see Fig. 9). Xib_1_ sequences (with their eight-cysteine scaffold similar to xib_13_) constitute compared to xib_13_ a more distinct, strongly supported clade (97 fast bootstraps); see Fig. 9.

Surprisingly, our phylogenetic analysis demonstrates that xib_2_, the ten-cysteine scaffolded xibalbin limited to *X. tulumensis*, emerges from the eight-cysteine scaffolded xib_1_ by an apparent gene duplication. The result is an ancestral, transitional xib_2_ sequence (c149924g1i1) with only eight and not ten cysteines which is highly similar in its primary sequence to the other xib_2_ family members; see Fig. 9. Finally, we show that xib_3_ sequences are closer related to six-cysteine scaffold ICKs from hexapods which indicates a possible six-cysteine variant that occurred in the ancestor of remipedes and hexapods. This result is in line with the findings of Maxwell et al. [56], who propose that xib_3_ derives from a domain duplication of an ancestral six-cysteine precursor that has since been lost in *X. tulumensis.* The findings in our phylogenetic analyses, especially the origin of xib_2_ from xib_1_, are as well supported in a CLANS analysis using pairwise sequence similarity clustering; see “Methods” and Additional File 10: Figure S6.

### Machine learning analysis largely corroborates the phylogenetic analysis

**Fig. 10:**
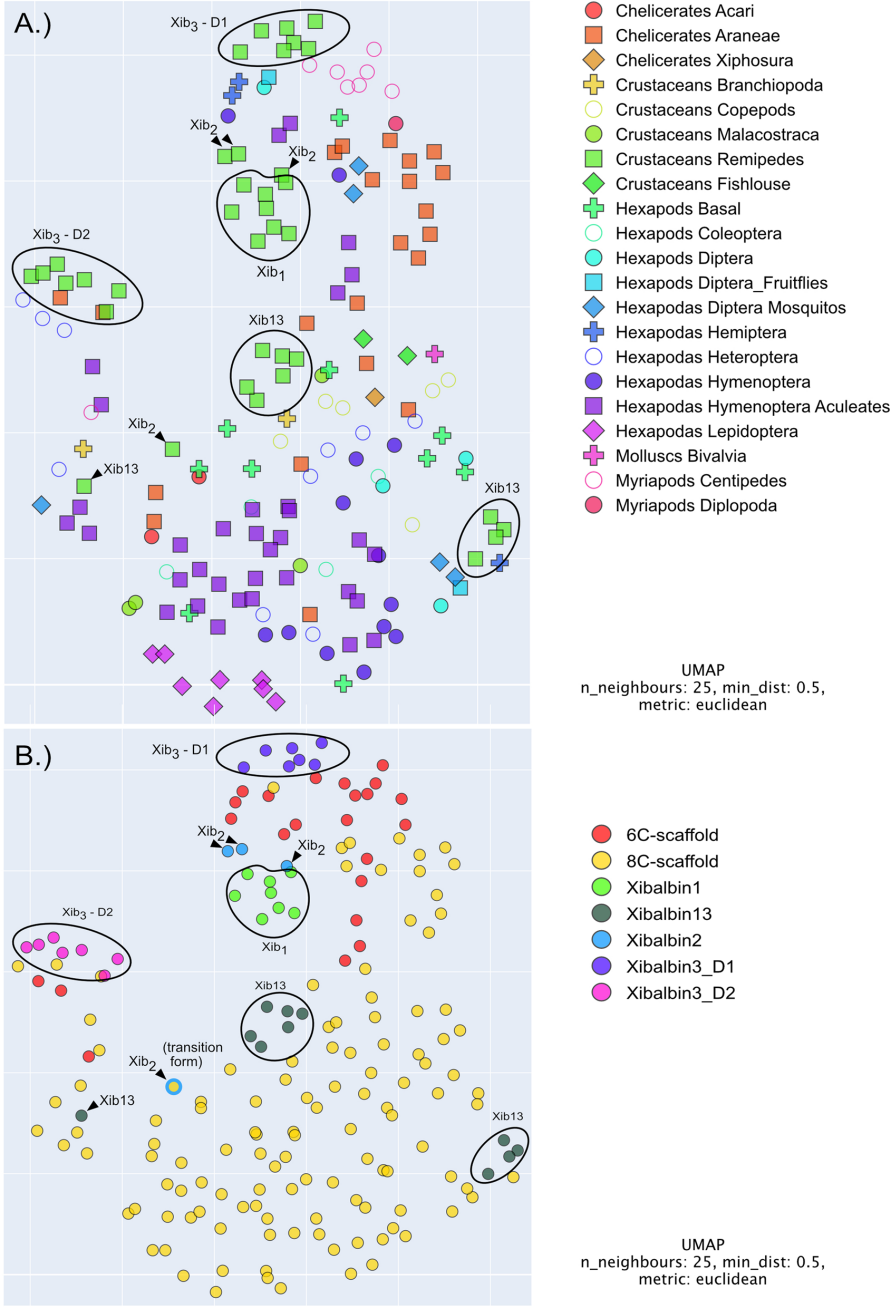
Machine learning generated protein space representations of xibalbins correspond with gene phylogeny-based grouping. All proteins corresponding with the sequences used to reconstruct the phylogenetic tree are color coded according to their major taxon clade (**A**) that are also indicated in the phylogenetic tree (Fig. 9) and additionally labeled according to their cysteine scaffold (**B**). See “Methods” for further details

To complement our phylogenetic analysis, we employed a novel machine learning (ML) method that constructs a multidimensional space of ICK relationships. This approach utilizes protein language models to generate a 1024-dimensional representation of proteins, known as “protein embeddings” [57]. These embeddings capture similarities based on the model’s understanding of protein structure and function, similar to how natural language processing understands text and predicts the probability of words appearing in a specific order. This method considers not only the sequence of amino acids but also their positions and interactions, even those separated by longer stretches, and captures nuances in structural and functional properties that are not apparent in direct sequence comparisons. It thus recognizes evolutionary relationships and functional classes of proteins without relying on “traditional” sequence alignment techniques that are based on positional homology; hence, it is termed “sequence independent”. We have to note though that the actual evolutionary process is more complex than what can be shown in visual representations due to the vast number of possible functional ICKs and the constraints of each lineage’s inheritance. For visualization purposes, we condensed this complex space into 3D, with 2D representations used in our figures (Fig. 10).

Our results largely corroborate the phylogenetic findings. Notably, xib_13_ sequences appear as the most ancestral within the remipede-specific cluster, given that xib_13_ represents a subfamily within a larger arthropod ICK subgroup. Furthermore, other remipede ICKs are positioned as close neighbors to xib_13_, reinforcing this interpretation.

Regarding xib_2_, our analysis revealed an intriguing bifurcation. While one form of xib_2_ clusters with other arthropod ICKs, its isolated positioning casts doubt on the significance of these associations. Interestingly, that sequence is the transitional xib_2_ sequence with only eight cysteines, which could mean that it exhibits as well a different or intermediate function that is more similar to xib_13_ resulting in this clustering pattern. In contrast, another variant of xib_2_ with ten cysteines is near the xib_1_ cluster, forming a distinct and tight grouping. This observation provides strong support for the close relationship between xib_1_ and xib_2_ as suggested by our phylogenetic analysis.

Lastly, the xib_3_ sequences displayed a clear clustering pattern by domain. Domain1 sequences of xib_3_ were found in close vicinity to a dense cluster of centipede ICKs. This arrangement suggests a potential evolutionary trajectory for xib_3_, likely originating from a centipede ICK and undergoing subsequent domain duplication.

## Discussion

We discuss here predominantly the examined activities of the xibalbin variants in the context of their potential pharmacological application and give first insight on their origins and evolution.

### Ion channel activity

Among the tested xibalbin variants, xib_13_ exhibited the most significant inhibition rates on voltage-gated potassium (Kv) and sodium (Nav) channels (Fig. 6). Intriguingly, despite their broad effects on Kv and Nav channels, none of the xibalbin variants affected T-type voltage-gated calcium channels (Cav3.x); see Fig. 5. Other examples of toxins acting on both sodium and potassium channels come from different species. For example, Nc1a, derived from *Nephila clavata* spider venom, acts on Nav and Kv channels in cockroach dorsal unpaired median neurons [58]. Similarly, HCTx from *Heteractis crispa* sea anemone venom shows remarkable target promiscuity [59, 60].

Both ion channel families (Kv and Nav) were inhibited by xib_1_ and xib_13_ with a preference towards voltage-gated potassium channels and a higher percentage of inhibition when compared to voltage-gated sodium channels. Xib_1_ and xib_13_ inhibit especially Kv1.1 channels which are important in controlling neuronal excitability as they are abundantly expressed in the nervous system [61]. In certain neuroinflammatory diseases such as multiple sclerosis and spinal cord injury, Kv1.1 channels show an altered axonal localization at the juxtaparanodal sites and hereby prevent electrical conduction along the neurons. It is reported that inhibiting Kv1.1 channels results in a reduction of pathological manifestations [62, 63].

In addition, xib_2a_ and xib_2b_ interacted primarily with Kv channels (Kv1.1, Kv1.2, Kv1.3, and Kv1.6) with only Nav1.4 being weakly inhibited by xib_2a_. The higher and more specific inhibition of Kv1.6 compared to other Kv channels by both Xib_2_ variants has direct application potential. Elevated Kv1.6 channel expression is known to be associated with pathological neuronal conduction in epileptic variants of epilepsy. The challenge so far has been that toxins that act on Kv1.6 channels also cross-react with other Kv channel types [64]. The characteristics of xib_2_ suggest that this toxin, perhaps with some synthetic modifications, could be of interest for applied studies of Kv1.6. From a biological perspective, xib_2_ is among the most highly expressed venom components in *X. tulumensis*, which implies its functional importance linked to the venom biology that remains, however, speculative. Interestingly, similar Kv channels are affected by all xibalbin variants (with the difference that no and only one Nav channel is affected by xib_2a_ and xib_2b_), despite their rather different primary sequences. A possible explanation for this phenomenon could be the close phylogenetic relationship between xib_1_ and xib_2_. We will discuss later that xib_2_ originates from xib_1,_ which could suggest that xib_2_ might have been adapted to increase the quantity of Kv-inhibiting toxins in remipede venom.

Interestingly, no xibablin showed inhibitory activity on *Shaker*, an insect Kv channel from *Drosophila melanogaster*; only mammalian voltage-gated potassium channels were affected. On the other hand, besides the activity recorded on mammalian voltage-gated sodium channels, BgNav, an insect Nav from the cockroach *Blattella germanica*, was targeted by Xib_1_ and Xib_13_, being the most affected Nav channel by the latter.

It was recently shown that the double ICK domain-like xib_3_ (which was not part of this study) targets RyR channels and promotes calcium release [56]. This suggests that calcium channel targeting is complementarily accomplished by xib_3_. Based on this new data, we speculate that the activities on ion channels might be divided among the xibalbins and that they act synergistically: xib_1_, xib_13_ (on Kv and Nav) xib_2_ (on Nav), and xib_3_ (on RyR). It is important to note though that the folded structures of all of the variants that are tested so far are approximations of the naturally secreted and folded xibalbins, providing first insight into their likely bioactivity. Obtaining crude venom to fractionate the toxins and reveal their natural conformation and bioactivity remains a challenge due to the rarity of remipede individuals in their remote habitats. Although we see no indication of diminished activity, we must note that we could not perform washout or concentration–response experiments to safely exclude artifacts. Nonetheless, the activity of the synthesized variants shows some promising activities for applied research that should be investigated further.

### Nociceptive neuron activity by *xib*_1_ and *xib*_13_

Sensory neurons exposed to xibalbins did not change in cell number, which indicates the absence of direct cytotoxic effects on cells (Additional File 7: Figure S5). Size and UCHL1 distribution of cells also remained the same among the tested groups suggesting there is no sub-group specific or neural cell death related to our xibalbin variants (Additional File 4: Figure S3). However, this is in line with other studies that test different ICK peptides such as GTx1-15, which has also been shown not to exert cytotoxicity in human cell lines even at high concentrations [65]. The recorded effects on ion channels regulating neuronal membrane potentials makes it interesting to look into the effect of the xibalbin variants on neurons, and we tested their activity on cultured sensory neurons. We observed increased PKA-II and pErk1/2 activity by xib_1_ and xib_13_ but not by xib_2_. This is in line with our data on the ion channel activity because the inhibition of Kv channels by xib_1_ and xib_13_ can prolong the action potential of the neuron, which then leads to activation of PKA-II and Erk1/2. Thouta et al. [66] reported that mice lacking the Kv1.1 coding gene show higher neuronal hyperexcitability, which is in agreement with our data showing that a more potent Kv1.1 inhibitor is a stronger activator of PKA-II and Erk1/2 (Fig. 8C-F and Fig. 8C-D). Whether such an activation is solely driven by the effects on membrane potential-regulating ion channels is not clear. There may also be an additional metabotropic activity of xib_1_ and xib_13_ similar to toxins such as α-latrotoxin or α-Bungarotoxin [66, 67].

We evaluated then the sensitization of rat DRGs by xib_1_ and xib_13_. Analysis of the size of cells reveals that mainly small-sized cells are responding to xibalbin toxins meaning that mostly nociceptive neurons are their predominant target (Fig. 8 G, H). As PKA and ERK activity has been characterized to result in pain behavior [68–71], our results imply that xib_1_ and xib_13_ are able to induce nociceptive neuron activity and are thus potential candidates for pain treatment. It should be noted that the concentrations used were higher than those used in the electrophysiological tests and caution should be exercised in making direct comparisons and biological interpretations. We can only speculate if this activity might reflect predatory or defensive functions of the natural xib_1_ and xib_13._ There is evidence that GPCR and RTK signaling already emerged in unicellular ancestors of metazoan and bilaterian species [72, 73]. In higher organisms, for example in *Aplysia*, GPCR signaling activating the cAMP/PKA pathway has been shown to regulate nociception [74] and the contribution of PKA and ERK to nociceptor hyperexcitability has been demonstrated [75, 76]. These studies suggest that the tested rat DRGs could theoretically reflect also possible activity in prey or predators of *X. tulumensis* [77–79].

### Evolutionary perspective on xibalbins

Our phylogenetic analysis illuminates first the possible origins of xib_13_, which is present in all five remipedes in a well-supported clade. Closely related to xib_13_ are sequences from early hexapod lineages, indicating that an older xib_13_ variant existed already in the common ancestor of hexapods and remipedes. More distantly related sequences are from non-venomous crustaceans (malacostracans, notostracans) and chelicerates; however, our topology is not sufficiently resolved in the deeper, more ancestral nodes to draw here further conclusions. Therefore, we can formulate two possible hypotheses on the deeper origin of xib_13_: either a common ancestral variant already existed in the ancestor of pancrustaceans and chelicerates, or xib_13_-like proteins evolved convergently in pancrustaceans and chelicerates. Given the clustering of protein embeddings of xib_13_ with other arthropodan ICKs in the machine learning analysis of protein space, the former scenario is more likely.

Xib_1_ sequences occur in all remipedes except *G. frondosus*. Nevertheless, it appears that xib_1_ is a common venom component in remipedes that is more unique to this group, which is reflected in the highly supported clade with remipede-only sequences (Fig. 9) and the protein space clustering (Fig. 10). Given the highly similar eight-cysteine scaffold of xib_1_ and xib_13_, our original hypothesis was that these two peptides are the result of a gene duplication that is followed by subsequent adaptation and high divergence of the sequences within the remipede lineage. However, both phylogenetic and machine learning results reveal separated monophyletic clades for both and thus rather support a separate origin probably from older gene duplication (Figs. 9 and 10). Xib_1_ experienced conservatively estimated at least two duplication events as seen in Fig. 9 (two different variants in *Pleomothra*); however, without genome data, this is difficult to interpret.

Surprising is our finding that one duplication event of xib_1_ within *Xibalbanus* leads to the origin of xib_2_* with an ancestral transitional form* (c149924g1i2) that has still eight cysteine patterns similar to xib_1_; see Figs. 2, 9, and 10*.* The primary sequence of this ancestral xib_2_ is highly similar to the other xib_2_ members but shorter. To draw a certain evolutionary scenario for this finding is somewhat challenging. In general, two novel cysteines could trivially appear by coupled point mutations in the sequence as it was shown in other venom proteins. For example, snake venom phospholipases A2 often evolve novel cysteines for polymerization, while three-finger toxins have evolved a novel inner bond [80, 81]. However, the caveat of this hypothesis is the shorter sequence. A less parsimonious hypothesis is that the duplication of the c149924g1i1 xib_1_ gene variant resulted in domain gain that added a sequence stretch in which two further cysteines evolved later. However, without genomic data, any hypothesis remains quite speculative also because the genomic processes that lead to the evolution of ICK peptides and their cysteine scaffolds are not yet studied in detail. Nevertheless, the origin of xib_2_ must underlie an important evolutionary constraint. It is a unique component of the venom gland secretion, implying that it is recruited and expressed exclusively in the venom system [27]. However, its high expression (fourth highest expressed venom component in *X. tulumensis*) suggests that it may be functionally more important than xib_13_ and xib_1_. The reasons for this, such as an adaptation to prey or predator, remain to be uncovered.

**Fig. 11:**
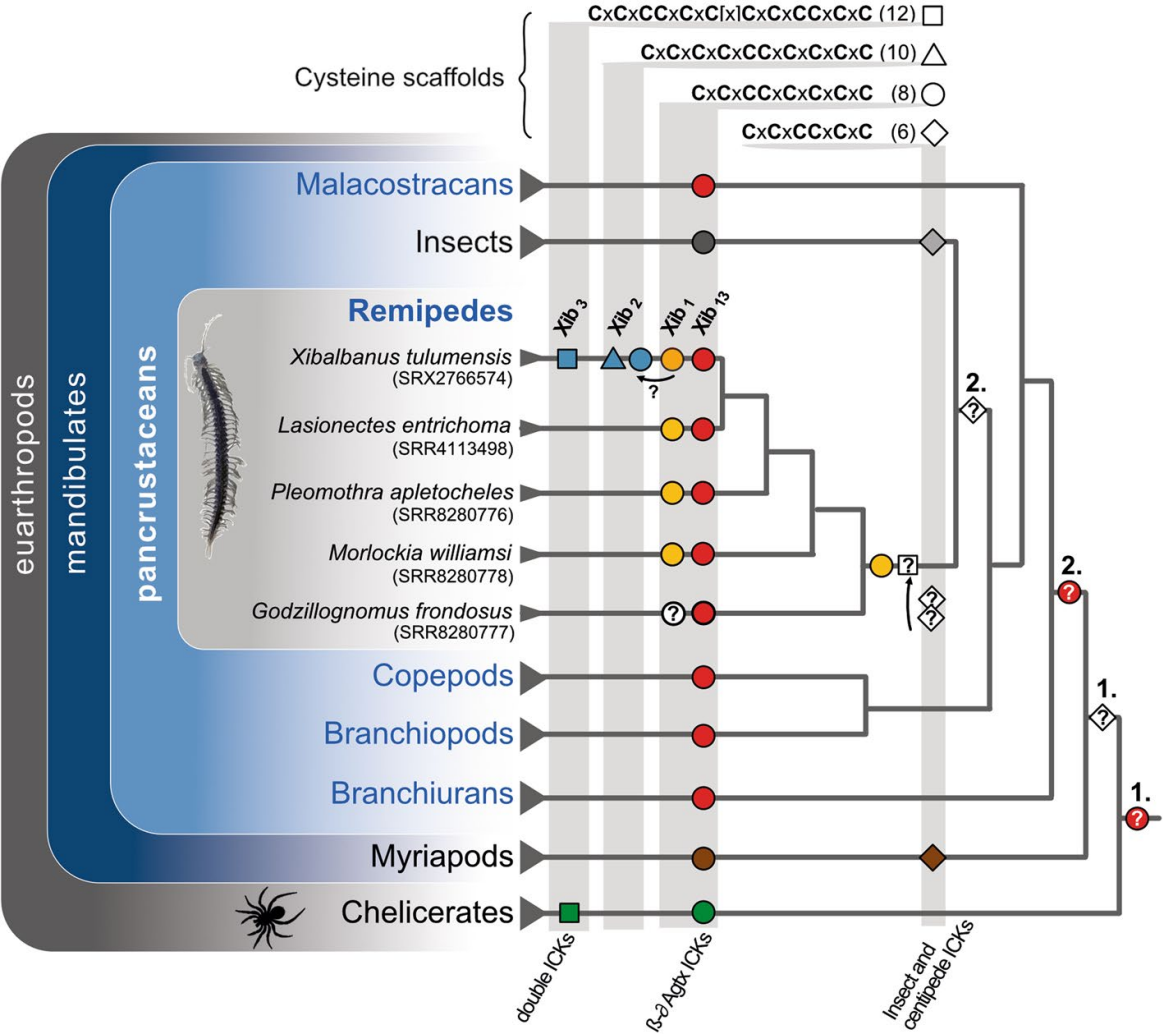
Synopsis of the evolution of xibalbins. Xib_1_ (yellow circle), xib_13_ (red circle), xib_2_ (blue triangle), and xib_3_ (blue square) in remipedes are depicted. Sequences with a similar cysteine scaffold (number of cysteins given in brackets) found in insects, other crustaceans, and remaining arthropods are illustrated as well in the same shape but different taxon-specific colors (chelicerates = green, myriapods = brown, insects = gray). Six cysteine scaffolds that are not found in remipedes or crustaceans are shown in the shape of a rhombus. Possible ancestral variants are indicated with a question mark and numbers on top for different alternative hypotheses. The phylogeny is taken from the most recent phylogenomic analysis of pancrustaceans [34]

Less expressed but also *Xibalbanus*-specific is xib_3_, which was not tested in this study but by Maxwell et al. [56]. We support their findings that the two six-cysteine scaffold domains of this double (domain) knottin are closely related to six-cysteine ICK sequences from insects (Figs. 10 and 11). Maxwell and colleagues argue that they evolved by duplication from an ancestral variant that is lost in *Xibalbanus*. We do not reject this hypothesis, however, given our results, it also implies the loss of six-cysteine variants in all other remipedes and crustaceans. However, our topology is not fully resolved in this part and we refrain from further conclusions.

To better understand the origin and remarkable convergent evolution of ICKs as highly adaptive peptides among major arthropod groups such as insects, crustaceans, and chelicerates, an extended analysis incorporating new proteo-transcriptome venom data from additional remipede species would be advantageous. It is equally crucial to obtain genome data from these arthropods, as recent research has demonstrated that solely utilizing proteo-transcriptome data in addressing gene-specific inquiries including gene origin and duplication can be flawed [23, 24, 82, 83]. Xibalbins are fundamental ICK-like peptides from an evolutionary viewpoint, providing insights into the emergence and evolution of this diverse toxin category, primarily in remipedes and hexapods, but also among other arthropods (Figs. 9, 10, and 11).

## Conclusions

Although structure and function of naturally occurring single domain xibalbin fractions from remipede venom remain to be to explored, our results based on synthesized variants support first functional insights that they affect predominantly potassium channels in combination with induced pain. Our results also show how important genome data will be to untangle the origin of xibalbins as part of the ICK-like venom protein family. As remipedes are the sister group to insects, the future identification of their ICK-like venom genes, along with an initial depiction of their conceivable mode of action and their phylogeny, is a crucial stepping stone to better understand the function and evolution of this hyper-diverse toxin group in remipedes and pancrustaceans.

## Methods

### Prediction of mature sequences of xibalbin variants and chemical synthesis

From the previous proteo-transcriptomic venom analysis of *X. tulumensis* xib_1_ and xib_2_ transcripts that are highly expressed and supported on proteome level by MALDI mass spectrometry were used as core sequences for this study [27, 84]. The two xib_1_ and xib_13_ transcripts (c27367_g1_i2_2 and c29168_g1_i2_VG_0) show high sequence similarity with known ∂-omegatoxin, which are knottins found in funnel web spiders with an ICK motif based on 8C residues [26, 85]. For xib_2_, only one higher expressed transcript (c29772_g1_i1_trinity_VG_ASS_5) was validated on the proteome level. Xib_2_ peptides feature an unusual 10C scaffold knottin-like sequence. Currently, no detailed analysis and structural conformation is known for single-domain xibalbins. We predicted the mature sequences of xib_1_ and xib_13_ by aligning these proteins with a representative highly similar structurally known ∂-omega toxin peptide from spider venom [85] that was already included in von Reumont et al. (2017) [27, 84] using EMBOSS secondary structure and cleavage site-packages (V1) within the software package Geneious Prime (2022.2.2); see Fig. 2. The domain area of xibalbin_2_ was predicted by aligning all transcript variants recovered in the proteo-transcriptomics study [27] and by using the EMBOSS secondary structure and cleavage site packages (V1) within the software package Geneious Prime (2022.2.2). We test here two variations (different cleavage sites) of these sequences with differing lengths of the mature protein; see Fig. 2.

### Evaluation of disulfide bonds by mass spectrometry

All chemically synthesized peptides were ordered at Vivitide, Gardner, MA, USA, performing the in-house solid-state synthesis by a standard automated peptide synthesizer. A non-directed refolding process using reduced/oxidized glutathione established thermodynamically stable disulfide bridges. In-house HPLC was run to evaluate the purity for all compounds with the following results: xib_1_ = 88.0%, xib_12_ = 84.9%, xib_2a_ = 99.9%, and xib_2b_ = 74.4% (Additional File 1: Figure S1). To verify sequence and structure, we sequenced all final xibalbin variants by bottom-up mass spectrometry analysis as digested peptides and as intact peptides (top-down). All xibalbin peptide sequences and purity (background noise of non-target fragments) were confirmed by tandem mass spectrometry (MS/MS) of the most abundant peptide fragments (Additional File 11: Figure S8).

The lyophilized undigested peptides were dissolved in ultrapure water (10 mg/mL), and either directly set to final concentration (2 mg/mL) with acidified ammonium bicarbonate buffer (25 mM ABC, 0.1% TFA, pH 7.8) for intact peptide mass analysis, or previously reduced (10 mM dithiothreitol), alkylated (20 mM iodoacetamide), and finally digested (15 ng/µL trypsin, Promega, Madison, United States) for bottom-up peptide analysis. In addition, native xibalbin variants were treated by either reduction (10 mM DTT) and alkylation (20 mM IAC) or direct digestion (15 ng/µL trypsin, Promega, Madison, USA). The matrix was prepared using a saturated stock solution of α-cyano-4-hydroxycinnamic acid (CHCA) in acetonitrile/ultrapure water (70% acetonitrile with 0.1% trifluoroacetic acid (TFA)). First, samples were spotted by dried droplet onto the ground steel target plate and matrix solution added on top. Mass analyses were performed on a MALDI-ToF/ToF mass spectrometer (ultrafleXtreme, Bruker Daltonics, Bremen, Germany) with the following operating settings: ion source 1 = 20.00 kV, ion source 2 = 17.75 kV, lens voltage = 8.00 kV, reflector voltage = 20.80 kV, optimized pulsed ion extraction time = 170 ns, matrix suppression = 600 Da, sample rate 5.00 GS/s, analog offset 68.90 mV, and positive reflectron (bottom-up peptide identification) or linear (intact peptide identification) mode. Laser strength and pulse ion extraction time were initially optimized and performance was calibrated using the calibrant peptide standard mixture. Peptides from mass spectra of in-solution digest samples were manually matched against the xibalbin amino acid sequences.

Identification of masses for refolded xib_1_ (5163.98 Da), xib_13_ (5521.05 Da), xib_2a_ (6202.69 Da), and xib_2b_ (5660.60 Da) variants, as well as of xibalbins, whose disulfide bridges were reduced and subsequently alkylated, (xib_1_ (5630.35 Da), xib_13_ (5987.25 Da), xib_2a_ (6784.21 Da), and xib_2b_ (6241.37 Da)) validated the correct number of disulfide bridges by the respective mass difference (Additional File 12: Figure S9). However, we have to note that the exact disulfide connectivity was not determined and that the synthetic peptides may not represent the natural peptides. All mass spectrometry proteomics data (.fid files) have been deposited via the MassIVE partner repository [86] under project name “Non-cytotoxic xibalbin ICK variants from remipede crustaceans” with the data set identifier MSV000091677 [87].

### Electrophysiological assays to test for ion channel activity

For the expression of K_v_ channels rat K_v_1.1 (GenBank accession number: NM_173095 [88, 89]), rK_v_1.2 (NM_012970 [90, 91]), human K_v_1.3 (NM_002232 [92, 93]), rK_v_1.4 (NM_012971 [94, 95]), rK_v_1.6 (NM_023954 [96, 97]), hK_v_2.1 (NM_004975 [98, 99]), hK_v_3.1 (NM_004976 [100, 101]), rK_v_4.2 (NM_031739 [102, 103]), hKv10.1 (NM_172362 [104, 105]), hERG1 [Kv11.1, (NM_000238 [106, 107])], and *Shaker* IR [from *Drosophila melanogaster,* (NM_167595 [108, 109])], Na_v_ channels [rNa_v_1.2 (NM_012647 [42, 110]), rNa_v_1.4 (NM_013178 [111, 112]), hNa_v_1.5 (NM_198056 [113, 114]), mNa_v_1.6 (NM_001077499 [115, 116]), BgNa_v_ [from *Blattella germanica,* (DQ466887 [117, 118])] and the auxiliary subunits rβ1 (NM_001271045 [119, 120]), hβ1 (NM_001037 [121, 122]), and TipE [from *D. melanogaster,* (NM_079196 [109, 123])], and Ca_v_ channels hCa_v_3.1 [124, 125], hCa_v_3.2 [126, 127], and hCa_v_3.3 [128, 129] in *Xenopus* oocytes, the linearized plasmids were transcribed using the T7 or SP6 mMESSAGEmMACHINE transcription kit (Ambion, Austin, TX, USA). Mature female animals were purchased from Nasco (Fort Atkinson, USA) and were housed in the Aquatic Facility (KU Leuven) in compliance with the regulations of the European Union (EU) concerning the welfare of laboratory animals as declared in Directive 2010/63/EU. The use of *X. laevis* oocytes was approved by the Animal Ethics Committee of the KU Leuven with the license number P186/2019. Stage V–VI oocytes were collected from anesthetized female *X. laevis* frog as previously described [130], with the frogs anesthetized by placement in 0.1% tricaine solution (amino benzoic acid ethyl ester; Merck, USA). Oocyte microinjection was performed using a microinjector (Drummond Scientifc®, USA), with a programmed cRNA injection volume of 4—50 nL, depending on the channel subtype. The oocytes were incubated in ND96 solution (96 mM NaCl, 2 mM KCl, 1.8 mM CaCl_2_, 2 mM MgCl_2_, and 5 mM HEPES, pH 7.4), supplemented with 50 mg/l gentamicin sulfate.

Electrophysiological measurements were performed at room temperature (18–22 °C) using the two-electrode voltage clamp (TEVC) technique. Data were obtained using a GeneClamp 500 amplifier (Axon Instruments, USA), and Clampex9 software (Axon Instruments), responsible for data acquisition and storage. Glass micropipettes were produced using glass capillaries (borosilicate WPI 1B120-6) and drawn in a WPI (World Precision Instruments, USA) manual stretcher. The bath and perfusion solutions were either the previously described ND96 (Na_v_ and K_v_ channels) or calcium-free ND96 supplemented with 10 mM BaCl_2_ (Cav channels).

Whole-cell currents of oocytes were recorded 1 to 3 days after injection. Current and voltage electrodes were filled with 3 M KCl and their resistance was adjusted from 0.7 to 2.0 MΩ. Currents were sampled at 20 kHz (Na_v_ channels) and 10 kHz (K_v_ and Ca_v_ channels) and filtered using a four-pole Bessel low-pass Bessel filter, at 1 kHz for sodium, and 500 MHz for potassium and calcium, except for the hERG ion channel, in which the currents were filtered at 1 kHz. Leak subtraction was performed using a -P/4 protocol. K_v_1.x currents were evoked by 500 ms depolarizations to 0 mV followed by a 500-ms pulse to − 50 mV, from a holding potential of − 90 mV. K_v_2.1, K_v_3.1, and K_v_4.2 currents were elicited by 500 ms pulses to + 20 mV from a holding potential of − 90 mV. Current traces of Kv10.1 were elicited by 2 s depolarization to 0 mV, from a holding potential of − 90 mV. Current traces of hERG1 channel were elicited by applying a + 40 mV prepulse for 2 s followed by a step of − 120 mV for 2 s. Sodium current traces were evoked by a 100 ms depolarization to 0 mV, from a holding potential of − 90 mV. The current–voltage (IV) relationships were determined by 100-ms step depolarizations between − 90 and + 40 mV, using 5 mV increments. For Ca_v_ channels, current traces were elicited by 700 ms depolarizations to − 20 mV from a holding potential of − 90 mV. Current values were expressed as means ± SEM of at least three independent experiments. Differences in ionic currents between control and sample conditions were compared by one-way ANOVA, followed by Dunnet multiple comparisons test. The shifts in *V*_half_ of activation and inactivation of Nav channels were compared by one-way ANOVA, followed by Tukey’s multiple comparisons test. Differences were considered statistically significant when *p* < 0.01.

### High-content imaging assay for sensory neuron activity

Male Sprague Dawley rats (250–400 g, 8–16 weeks old) were obtained from Envigo. Rats were kept in 12 h of light/darkness cycles. All experiments were performed in accordance with the German animal welfare law with permission of the District Government for Nature and Environment, NRW (84–02.05.20.13.045, 4.18.003). Rats were scarified by slow inhalation of CO_2_ which was followed by decapitation. DRGs were extracted from rats, deheated, pooled, and incubated in Neurobasal A/B27 medium (Invitrogen, #12349–015) supplemented with B-27 (Invitrogen #17504), _L_-glutamine 1:400, _L_-glutamate 1:270.3, and penicillin/streptomycin 1:100 containing 0.2 U/mL collagenase P (collagenase P, Roche, #1213857) and incubated for 1 h at 37 °C, 5% CO_2_. DRGs were dissociated with pasteur pipettes and axon stumps were separated by BSA (Sigma-Aldrich, # A2153-100G) gradient centrifugation (14% BSA, 120 g, 8 min). Cells were resuspended in NBA medium, plated in 96-well imaging plate (Greiner BioOne Sensoplate, Black, #655896) precoated with poly-L-ornithine hydrochloride (0.1 mg/ml Sigma, #P2533)/laminin (5 µg/ml Invitrogen, #23017–015), and incubated overnight at 37 °C, 5% CO_2_.

DRGs were stimulated after overnight incubation. Compounds were prepared as tenfold concentrated stock solutions, diluted in PBS (PAA, cat# H15-002), in v-bottom plates; 50 µL media from culture wells were mixed with the 12.5 µL stock solutions, and 50 µL added back to the respective wells. Stimulation was performed with automated multichannel pipette and cells were kept in heated block during the stimulation. Cells were then fixed for 10 min at RT with paraformaldehyde (final concentration: 4%, Cat# 1.04005) at desired time points. Fixed cells were washed twice with PBS. Following blocking and permeabilization (2% normal goat serum (Dianova, Hamburg, Germany, #005‐000‐121), 1% BSA, 0.1% Triton X‐100 (Roth, Karlsruhe, Germany, #3051.2), 0.05% Tween 20 (Sigma‐Aldrich, #P9416)) for 1 h at RT, respective primary antibodies diluted in 1%BSA in PBS was added to the cells and incubated for and overnight at 4 °C. After that, cells were washed three times with PBS (10 min) and secondary antibodies (1:1000, fluorescently labeled) and DAPI (50 ng/ml-1) for 1 h at RT in dark. Finally, cells were washed three times with PBS (10 min) and wells were filled with PBS, sealed, and kept at 4 °C until scanning.

Stained cells were scanned with a CX7-LZR (Thermo Fisher Scientific) HCI system. Images were acquired with a 10 × objective and analyzed using the cellomics software package (Thermo Fisher Scientific). UCHL1 channel was used as a marker to identify neurons. Object selection was further based on the following criteria: 120–6000 µm^2^; circularity: 1–2; length‐to‐width ratio: 1–2; average intensity: 250–2000; and total intensity: 6 × 104 to 5 × 106. The resulting objects were quantified for average object intensity in all other color channels. Untreated wells were used for normalization and compensation was performed for minimizing spill over between channels. All analyses were conducted using R programming language and RStudio as integrated development environment (IDE). One-way ANOVA, Dunnett’s test post hoc was performed to evaluate statistical significance between groups. The difference between the two means (D) divided by the standard error of that difference (computed from all the data): *q* = D/SED.

### Identification of xibalbin1 and xibalbin2 variants and phylogenetic reconstruction

We then mined identified xibalbin sequences in whole-body transcriptomes of four other published remipede species [33, 40] to identify possible xib_1,_ xib_13,_ xib_2_, and finally xib_3_ sequence variants via an automated hmmer-search. In brief, available comprehensive alignments of all xibalbin sequences reconstructed from published *X. tulumensis* transcriptome data (SRX2766574) [27, 84, 131] were used to train hmm-models using the av-hmmer-pipeline [41]. The matching sequences were identified in the translated ORFs. Beforehand, the raw read data of the four remipede species were downloaded from the SRA archive (NCBI): *L. entrichoma* (SRR4113498) [40, 132], *M. williamsi* (SRR8280778) [33, 133], *G. frondosus* (SRR8280777) [33, 134], and *P. apletocheles* (SRR8280776) [33, 135]. The raw reads were quality checked with FastQC [136] and processed with Trimmomatic v0.38 [137] using standard settings except for a chosen quality threshold of phred 30 and a minimum length of 50 bp. The trimmed reads were assembled with Trinity v2.8.4 [138] using standard settings except for a minimum length of 100 bp (Additional File 13: Data S2, Additional File 14: Data S3, Additional File 15: Data S4, Additional File 16: Data S5). Open reading frames (ORF) with a minimum of 40 aa were predicted with Transdecoder v5.0.4 (Additional File 17: Data S6, Additional File 18: Data S7, Additional File 19: Data S8, Additional File 20: Data S9).

All sequences of xib_1_, xib_2,_ and xib_13_ alignments (Additional File 21: Data S10, Additional File 22: Data S11, Additional File 23: Data S12) were combined with known ICK toxins and highly similar sequences from non-venomous arthropods available in UniProt combining the sequences used in Maxwell et al. [56, 139] (with separated first and second double ICK domains of xib_3_) and von Reumont et al. [27]; see Additional File 24: Data S13. Signal peptide, propeptide, and mature regions were separately aligned for all sequences with optimization strategy for one domain using Mafft-L-INS-I [140] and then concatenated. The phylogenetic tree was reconstructed with IQ-TREE2 [141, 142] on 56 cores using settings for rapid bootstraps with integrated model fitting and branch length optimization (MFP, -B 25000, -bnni, -T 56). The original tree (Additional File 9: Data S1) was condensed for Fig. 9 by collapsing all nodes below a support value of 50. The complementary CLANS analysis was performed with standard settings using the Java version 29.05.2012 and 81,987 rounds [143].

### Construction of ICK embedding protein language model space

We leveraged modern advances in machine learning, in particular—in natural language models adopted to work with proteins—protein language models (or pLMs) [144]. These models have been successfully used to create protein space for various datasets, including our own work [23, 81]. It was shown that distance in embedding space correlates with protein function and can be used as an orthogonal signal for clustering proteins into functional families [145].

Here, we used the pLM ProtT5-XL-UniRef50 [144] (in the following ProtT5) to create fixed-length vector representations for each protein sequence (per-protein embeddings) irrespective of its length. To achieve that, we first created individual vector representations for each residue in a protein and then averaged over all residue embeddings in a protein to derive fixed-length vector representations for single proteins (per-protein embedding) irrespective of a protein’s length. As ProtT5 was only trained on unlabeled protein sequences and no supervised training or fine-tuning was performed, there is no risk of information leakage or overfitting to a certain class or label. As a result, every protein was represented as 1024-dimensional per-protein embeddings. Those high-dimensional representations were projected to 3D using UMAP (n_neighbors = 25, min_dist = 0.5, random_state = 42, n_components = 3) and colored according to their respective group to allow for visual analysis. Embeddings were created using the bio_embeddings package [145]. Interactive 3D plots of protein spaces are given in Additional File 25: Data S14 (proteins labeled according to taxa clades and protein families) and Additional File 26: Data S15 (proteins labeled according to cysteine scaffold) and were reconstructed using the algorithm deposited on github: https://github.com/Rostlab/RostSpace. 

### Supplementary Information

Additional file 1: Figure S1. HPLC spectrograms of all xibalbins. All information and retention times are given in the tables to the right of each spectrogram.

Additional file 2: Table S1. Individual data values of all electrophysiological experiments.

Additional file 3: Figure S2. Number of analyzed cells A) at 5 and B) at 30 min. At different concentrations. There is no significant change in number of cells against control (concentration 0). Statistics: One Way ANOVA, Dunnett's post hoc. Data represent mean ± s.e.m.

Additional file 4: Figure S3. A) Size and B) UCHL1 intensities of the tested cells among replicas, see Material and Methods. There is no difference on the size and UCHL1 intensities of the tested cells between 4 replicas. C) Size and D) UCHL1 intensities of the tested cells among different time points. Overall, there is no difference in the size and UCHL1 intensities of the tested cells among replicas and tested conditions.

Additional file 5: Figure S4. Effects of xibalbins on cytotoxicity in RAW264.7 macrophages and adhesion of leukocytes onto the vascular endothelium. (i) For the viability assay, RAW264.7 cells were treated with 25 μg/ml concentrations of xibalbins for 24 h. Cells were incubated with WST-8 and the formed formazan was detected by absorbance measurements. (ii) For the proliferation assay, HMEC-1 cells were grown in low density and treated after 24 h with the indicated peptide for 72 h. Cells were stained with crystal violet solution. The amount of DNA-bound crystal violet was detected by absorbance measurements. (iii) Xibalbins do not interfere with the adhesion of leukocytes on endothelial cells. THP-1 cell adhesion under static conditions. HMECs were grown to confluence, preincubated with xibalbins for 30 min, and activated with TNF (10 ng/ml) for 24 h. For the leukocyte adhesion assay, untreated THP-1 cells (3 × 10^4^ cells/well) were stained with CellTracker Green (Thermo Fisher Scientific, Frankfurt am Main, Germany) and were allowed to adhere to the treated HMECs for 5 min. The adhesion of leukocytes onto endothelial cells was quantified by fluorescence measurements using a Tecan Infinite F200 Pro microplate reader (Tecan, Männedorf, Switzerland) (excitation: 485 nm, emission: 535 nm). See Additional File 6 for all individual values.

Additional file 6: Table S2. Individual data values of all UCHL1, pRII, pERK1/2 HCIS experiments.

Additional file 7: Figure S5. A) For the induction assay (left panel) HEK293T cells were treated with 4 μM Fluo-8-AM in 100 µl HBSS for 1 h, 37 °C. Five images/sec were taken using an ImageXpress Micro Confocal High Content Imaging System. Xibalbins (0.25, 2.5, 25 μg/ml), DMSO (negative control), or 5 μM ionomycin (positive control) were added with images taken every second for 20 s. For the inhibition assay (right panel), the peptide-treated samples (30 min) were treated with 5 μM ionomycin with images taken every second for 20 s. MetaXpress Software Version 6 was used for data analysis. A threshold of fluorescence intensity was defined using cells before treatment, all cells above the threshold level were counted. The number of cells above the threshold in the toxin-treated samples was related to the cells in the DMSO- or ionomycin-treated sample. B) HEK293T cells were transfected with pGloSensor-22F cAMP plasmid (E2301, Promega, Walldorf, Germany) using turbofect reagent (Thermofisher Scientific, Frankfurt am Main, Germany). cAMP transfected HEK293T cells were incubated in DMEM without phenol red supplemented with pGlo sensor cAMP reagent (E1290, Promega, Walldorf, Germany). Induction and inhibition assay were performed in two steps with the same plate. For the induction assay (left panel), the luminescence was detected (background, 3 measurements every 5 min) and then the xibalbins (0.25, 2.5, 25 μg/ml) or 5 μM forskolin were added to detect the luminescence (3 measurements/5 min) using a plate reader (Spark, Tecan, Männedorf, Switzerland). For the inhibition assay (right panel), the xibalbin-treated cells were incubated with 5 μM forskolin to detect the luminescence (3 measurements/5 min). Luminescence values of xibalbin-treated samples were related to the DMSO- or forskolin-treated sample. C) For the induction assay (left panel), we treated the RAW264.7 macrophages with the xibalbins, DMSO and 100 ng/ml lipopolysaccharide (LPS) (positive control). For the inhibition assay (right panel) cells were 30 min pre-incubated with peptides or control (DMSO) before adding 100 ng/ml LPS. After 24 h NO was determined in supernatants with the Griess method. The NO levels of the xibalbin-treated samples were related to the DMSO- or LPS-treated sample. See Additional File 6 for all individual values.

Additional file 8: Table S3. Individual data values of all RAW246.7 and cytotoxicity experiments.

Additional file 9: Data S1. Phylogenetic tree file of all ICK peptides from remipedes and higher arthropods reconstructed in IQTREE.

Additional file 10: Figure S7. Results of the pairwise sequence similarity clustering analysis using standard setting in CLANS are shown.

Additional file 11: Figure S8. Peptide sequencing of xibalbin variants by MALDI-ToF/ToF mass spectrometry.

Additional file 12: Figure S9. Dilsulfide bond matching of xibalbin variants by MALDI-ToF/ToF MS.

Additional file 13: Data S2. New de novo assembly of the remipede transcriptome SRR4113498 generated with Trinity, as fasta file. (39.2 MB).

Additional file 14: Data S3. New de novo assembly of the remipede transcriptome SRR8280778 generated with Trinity, as fasta file

Additional file 15: Data S4. New de novo assembly of the remipede transcriptome SRR8280776 generated with Trinity, as fasta file.

Additional file 16: Data S5. New de novo assembly of the remipede transcriptome SRR8280777 generated with Trinity, as fasta file.

Additional file 17: Data S6. Open reading frames predicted with Transdecoder for the new remipede transcriptome assembly SRR4113498, as fasta file. (26.9 MB).

Additional file 18: Data S7. Open reading frames predicted with Transdecoder for the new remipede transcriptome assembly SRR8280778, as fasta file.

Additional file 19: Data S8. Open reading frames predicted with Transdecoder for the new remipede transcriptome assembly SRR8280776, as fasta file.c

Additional file 20: Data S9. Open reading frames predicted with Transdecoder for the new remipede transcriptome assembly SRR8280777, as fasta file.

Additional file 21: Data S10. All xibalbin_1_ sequences aligned with Mafft, as fasta file.

Additional file 22: Data S11. All xibalbin_2_ sequences aligned with Mafft, as fasta file.

Additional file 23: Data S12. All xibalbin_13_ sequences aligned with Mafft, as fasta file.

Additional file 24: Data S13. All xibalbin sequences including xibalbin_3_ and other arthropod ICK aliged with mafft, as fasta file.

Additional file 25: Data S14. All sequences in 3D space illustrating functional relations labeled according to taxon and protein family, html file.

Additional file 26: Data S15. All sequences in 3D space illustrating functional relations labeled according to cysteine-scaffold, html file.

## Data Availability

All data generated or analyzed during this study are included in the supplementary information files of this article, including the individual data values of all experiments (Additional File 2: Table S1, Additional File 6: Table S2, Additional File 8: Table S3). The transcriptome assemblies and protein predictions that were generated from published data (see references) are additionally provided in the open-access database ZENODO, 10.5281/zenodo.7808089 [146]. The proteome data to test the synthesized products is deposited in the MassIVE data repository MSV000091677 (https://massive.ucsd.edu/ProteoSAFe/dataset.jsp?task=058eae6afbb5402a987e9ca55312f8cc) [87].

## Abbreviations

ICK: Inhibitor cysteine knot
PKA: Protein kinase A
ERK: Extracellular signal-regulated kinase
FSK: Forskolin
LDLa: Low-density lipoprotein receptor domain class A
HCI: High content imaging
UHCL(1): Ubiquitin C-terminal hydrolase L(1)
DRG: Dorsal root ganglion
HMEC: Human mammary epithelial cells
ORF: Open reading frame
pRII: Phosphorylated regulatory subunit type
TEVC: Two-electrode voltage clamp
KCl: Potassium chloride
