# Supplementary figures and images for: Diversely evolved xibalbin variants from remipede venom inhibit potassium channels and activate PKA-II and Erk1/2 signaling
Source: BMC Biol. 2024 Jul 29;22:164. doi: 10.1186/s12915-024-01955-5 (PMC11288129; doi:10.1186/s12915-024-01955-5)

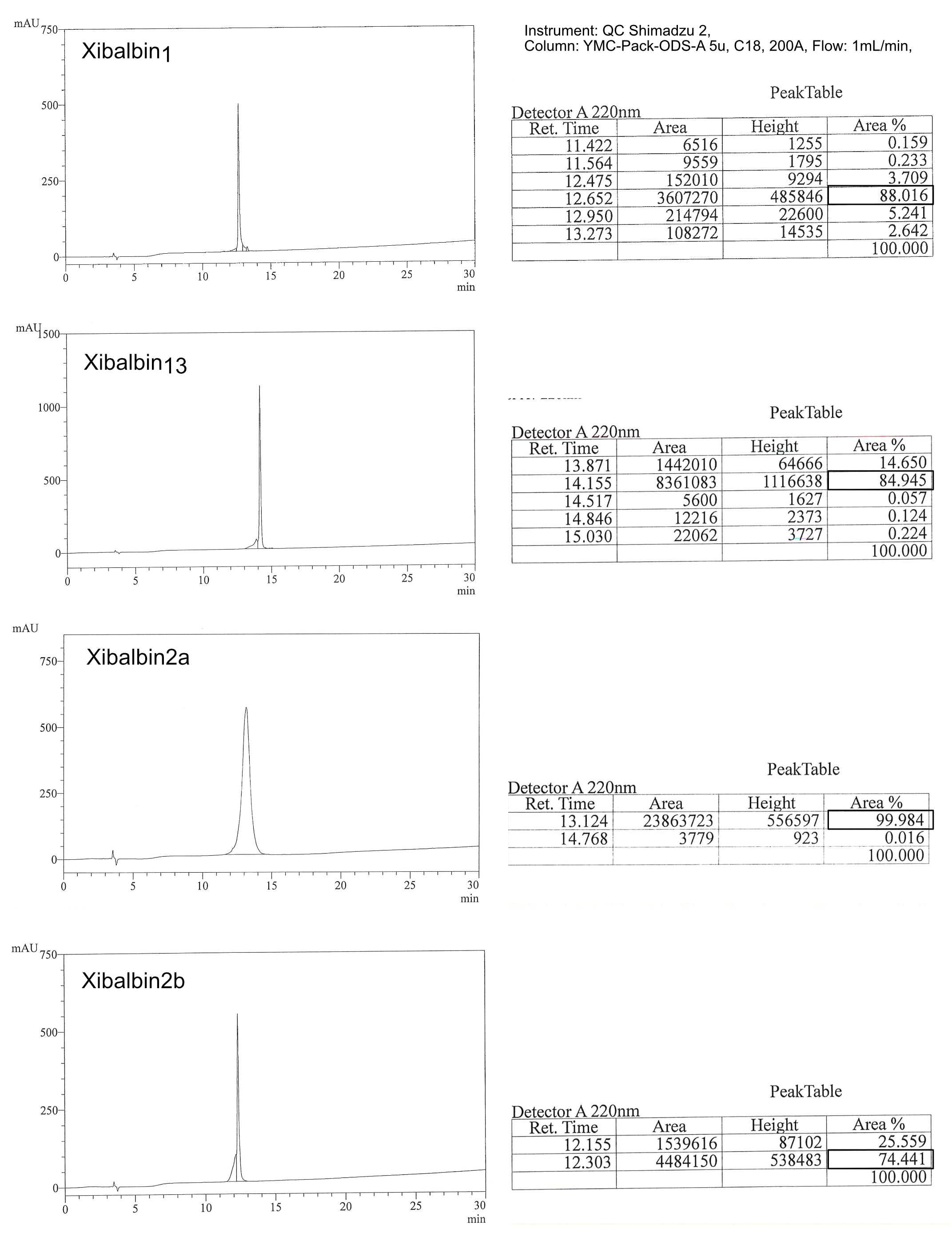

Supplement: Supplementary file 1 — Additional file 1: Figure S1. HPLC spectrograms of all xibalbins. All information and retention times are given in the tables to the right of each spectrogram. [file 12915_2024_1955_MOESM1_ESM.jpg]

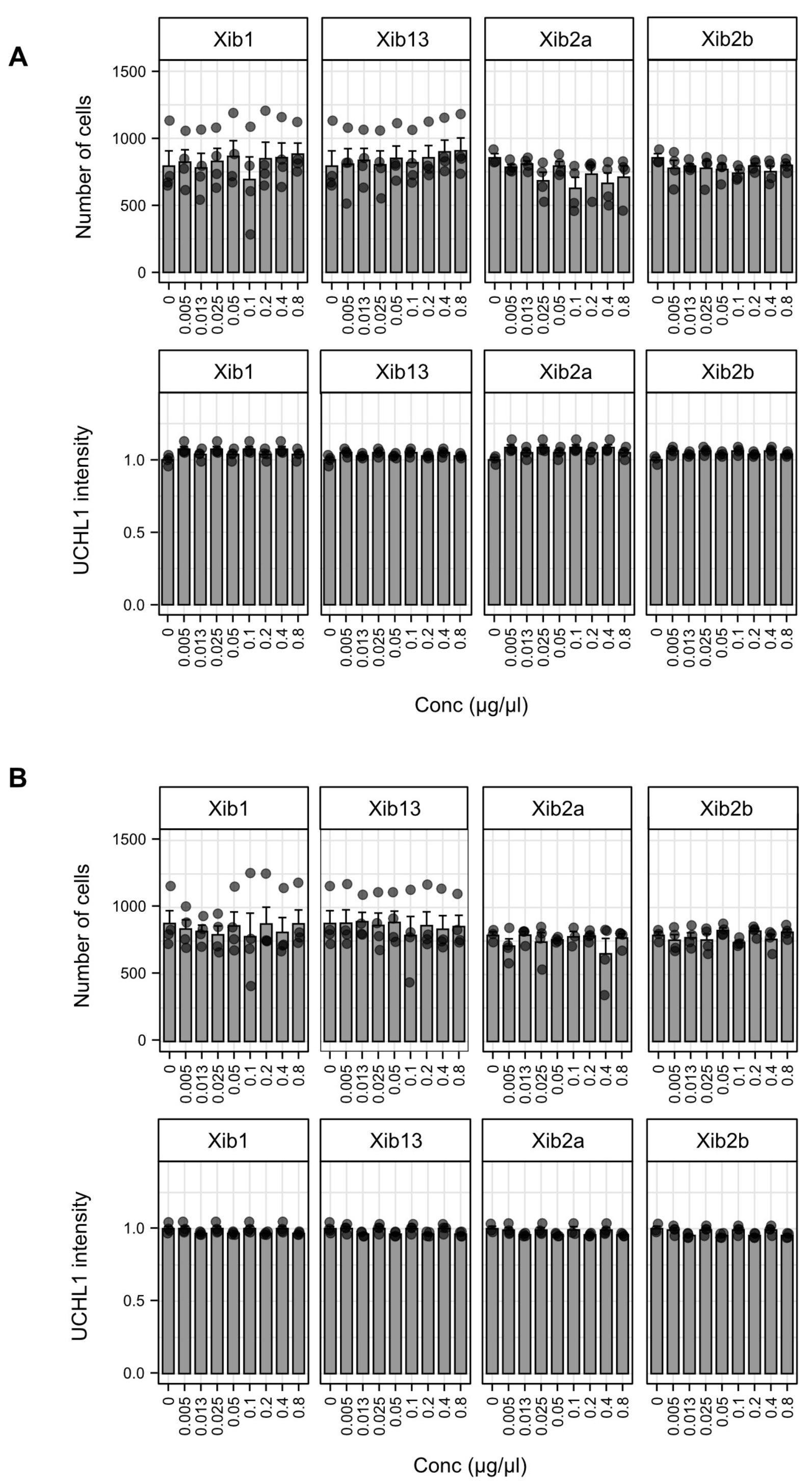

Supplement: Supplementary file 3 — Additional file 3: Figure S2. Number of analyzed cells A) at 5 and B) at 30 min. At different concentrations. There is no significant change in number of cells against control (concentration 0). Statistics: One Way ANOVA, Dunnett's post hoc. Data represent mean ± s.e.m. [file 12915_2024_1955_MOESM3_ESM.jpg]

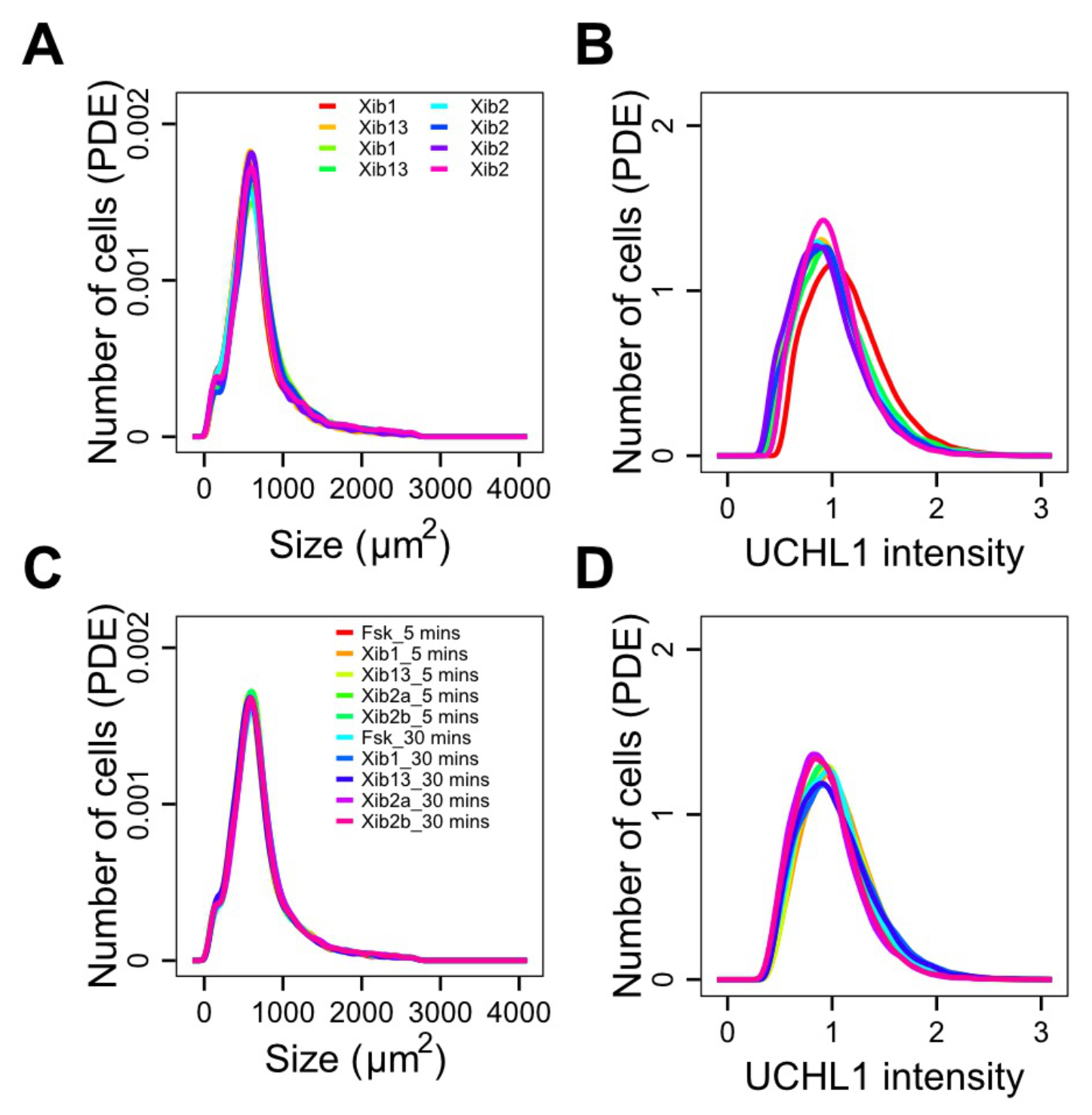

Supplement: Supplementary file 4 — Additional file 4: Figure S3. A) Size and B) UCHL1 intensities of the tested cells among replicas, see Material and Methods. There is no difference on the size and UCHL1 intensities of the tested cells between 4 replicas. C) Size and D) UCHL1 intensities of the tested cells among different time points. Overall, there is no difference in the size and UCHL1 intensities of the tested cells among replicas and tested conditions. [file 12915_2024_1955_MOESM4_ESM.jpg]

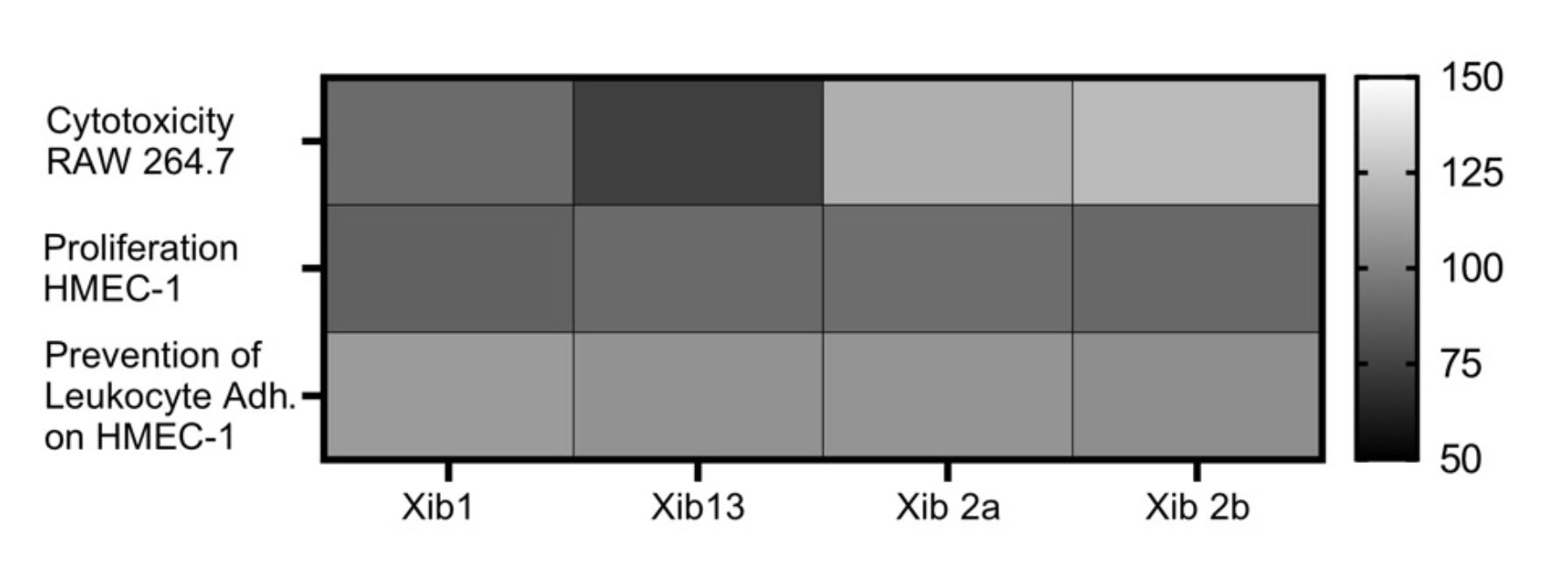

Supplement: Supplementary file 5 — Additional file 5: Figure S4. Effects of xibalbins on cytotoxicity in RAW264.7 macrophages and adhesion of leukocytes onto the vascular endothelium. (i) For the viability assay, RAW264.7 cells were treated with 25 μg/ml concentrations of xibalbins for 24 h. Cells were incubated with WST-8 and the formed formazan was detected by absorbance measurements. (ii) For the proliferation assay, HMEC-1 cells were grown in low density and treated after 24 h with the indicated peptide for 72 h. Cells were stained with crystal violet solution. The amount of DNA-bound crystal violet was detected by absorbance measurements. (iii) Xibalbins do not interfere with the adhesion of leukocytes on endothelial cells. THP-1 cell adhesion under static conditions. HMECs were grown to confluence, preincubated with xibalbins for 30 min, and activated with TNF (10 ng/ml) for 24 h. For the leukocyte adhesion assay, untreated THP-1 cells (3 × 10^4^ cells/well) were stained with CellTracker Green (Thermo Fisher Scientific, Frankfurt am Main, Germany) and were allowed to adhere to the treated HMECs for 5 min. The adhesion of leukocytes onto endothelial cells was quantified by fluorescence measurements using a Tecan Infinite F200 Pro microplate reader (Tecan, Männedorf, Switzerland) (excitation: 485 nm, emission: 535 nm). See Additional File 6 for all individual values. [file 12915_2024_1955_MOESM5_ESM.jpg]

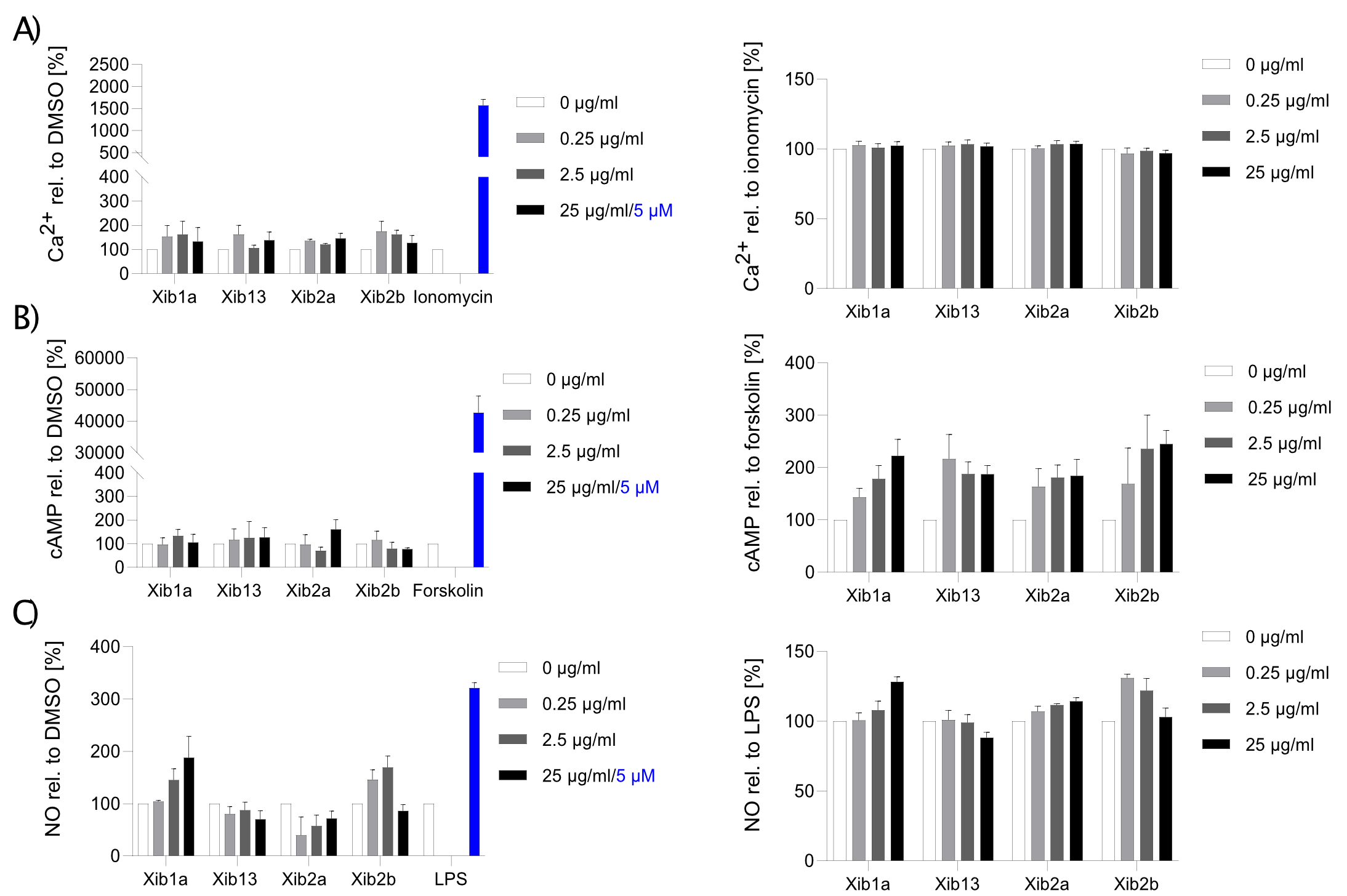

Supplement: Supplementary file 7 — Additional file 7: Figure S5. A) For the induction assay (left panel) HEK293T cells were treated with 4 μM Fluo-8-AM in 100 µl HBSS for 1 h, 37 °C. Five images/sec were taken using an ImageXpress Micro Confocal High Content Imaging System. Xibalbins (0.25, 2.5, 25 μg/ml), DMSO (negative control), or 5 μM ionomycin (positive control) were added with images taken every second for 20 s. For the inhibition assay (right panel), the peptide-treated samples (30 min) were treated with 5 μM ionomycin with images taken every second for 20 s. MetaXpress Software Version 6 was used for data analysis. A threshold of fluorescence intensity was defined using cells before treatment, all cells above the threshold level were counted. The number of cells above the threshold in the toxin-treated samples was related to the cells in the DMSO- or ionomycin-treated sample. B) HEK293T cells were transfected with pGloSensor-22F cAMP plasmid (E2301, Promega, Walldorf, Germany) using turbofect reagent (Thermofisher Scientific, Frankfurt am Main, Germany). cAMP transfected HEK293T cells were incubated in DMEM without phenol red supplemented with pGlo sensor cAMP reagent (E1290, Promega, Walldorf, Germany). Induction and inhibition assay were performed in two steps with the same plate. For the induction assay (left panel), the luminescence was detected (background, 3 measurements every 5 min) and then the xibalbins (0.25, 2.5, 25 μg/ml) or 5 μM forskolin were added to detect the luminescence (3 measurements/5 min) using a plate reader (Spark, Tecan, Männedorf, Switzerland). For the inhibition assay (right panel), the xibalbin-treated cells were incubated with 5 μM forskolin to detect the luminescence (3 measurements/5 min). Luminescence values of xibalbin-treated samples were related to the DMSO- or forskolin-treated sample. C) For the induction assay (left panel), we treated the RAW264.7 macrophages with the xibalbins, DMSO and 100 ng/ml lipopolysaccharide (LPS) (positive control). For the inhi [file 12915_2024_1955_MOESM7_ESM.jpg]

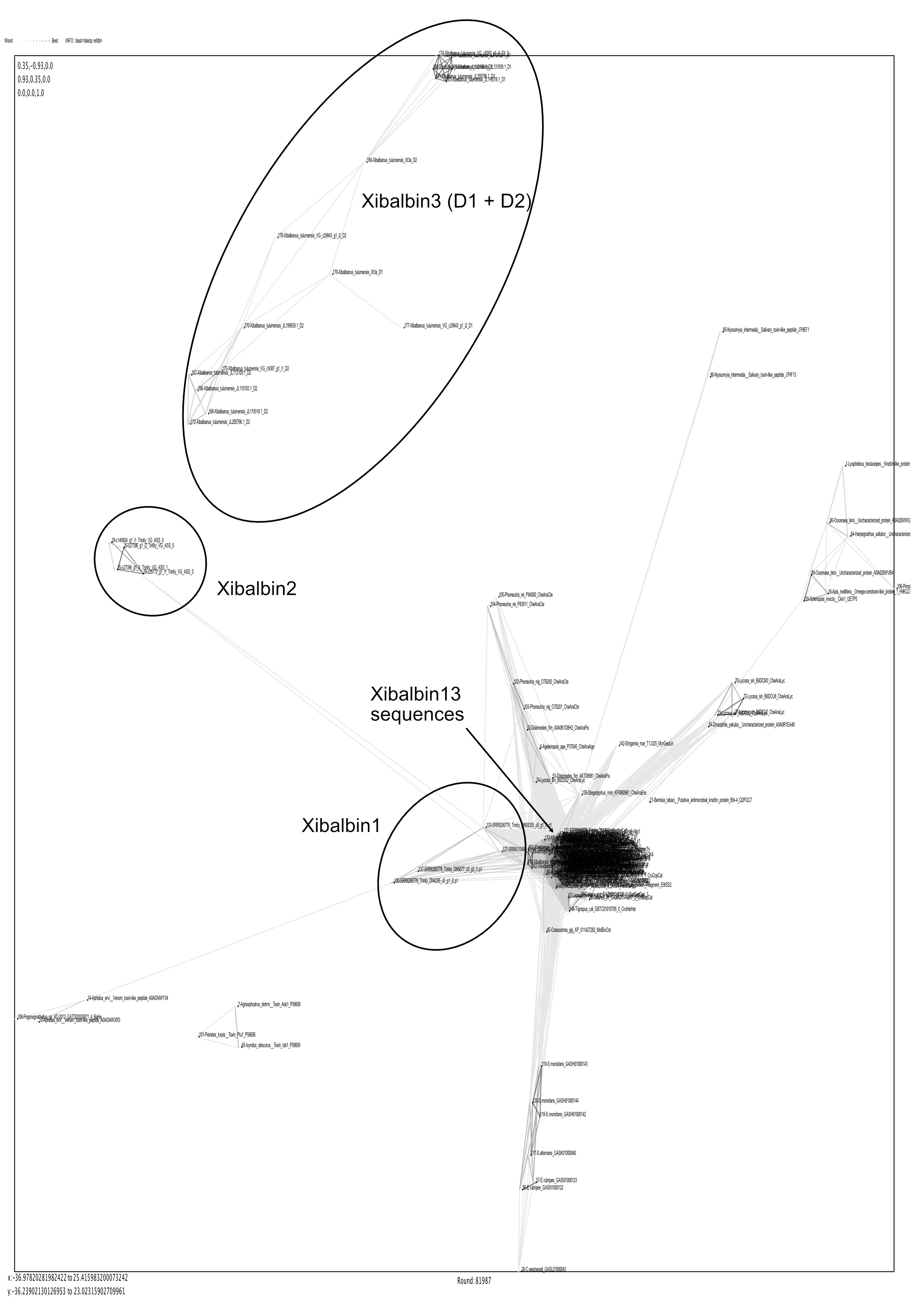

Supplement: Supplementary file 10 — Additional file 10: Figure S7. Results of the pairwise sequence similarity clustering analysis using standard setting in CLANS are shown. [file 12915_2024_1955_MOESM10_ESM.jpg]

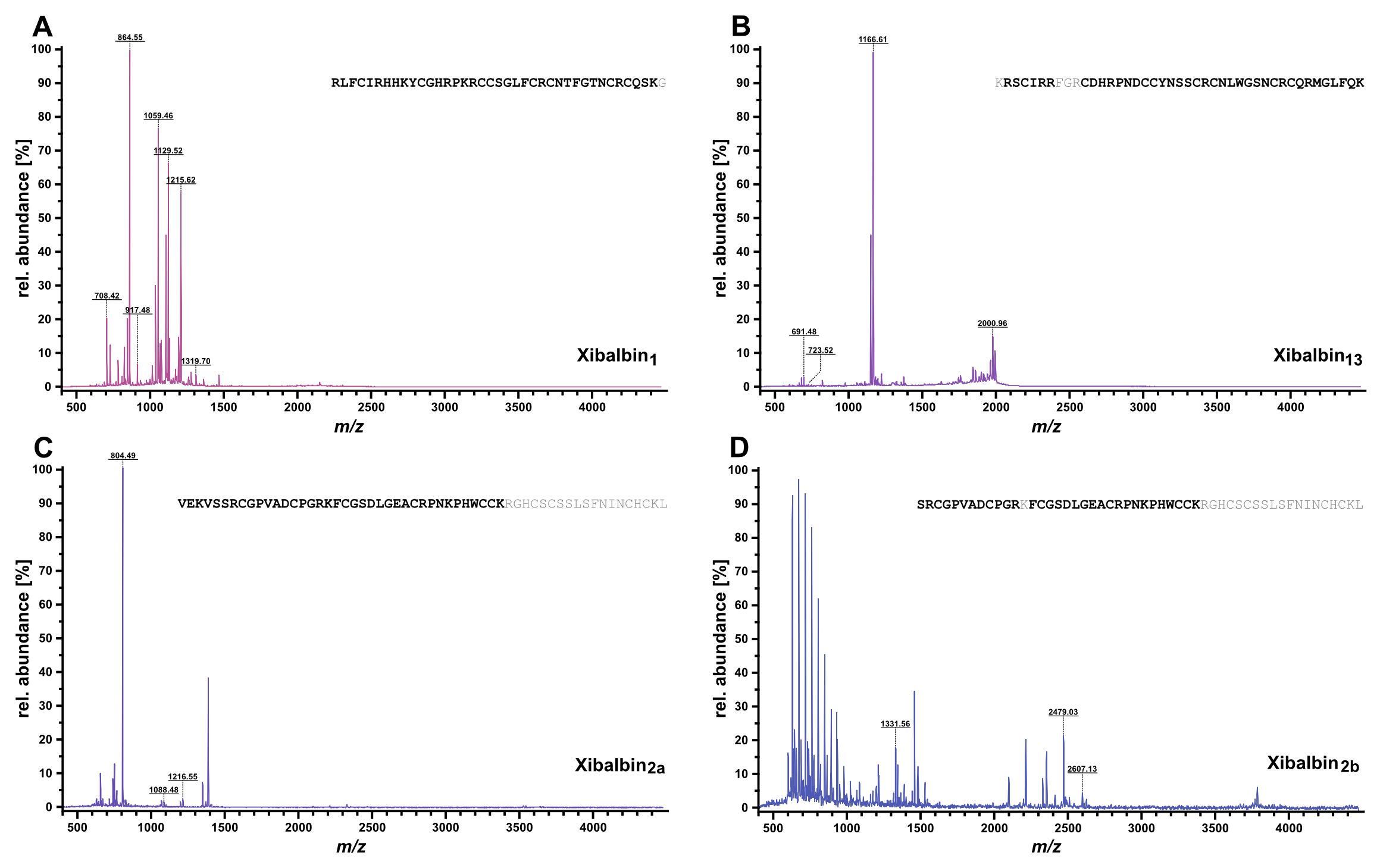

Supplement: Supplementary file 11 — Additional file 11: Figure S8. Peptide sequencing of xibalbin variants by MALDI-ToF/ToF mass spectrometry. [file 12915_2024_1955_MOESM11_ESM.jpg]

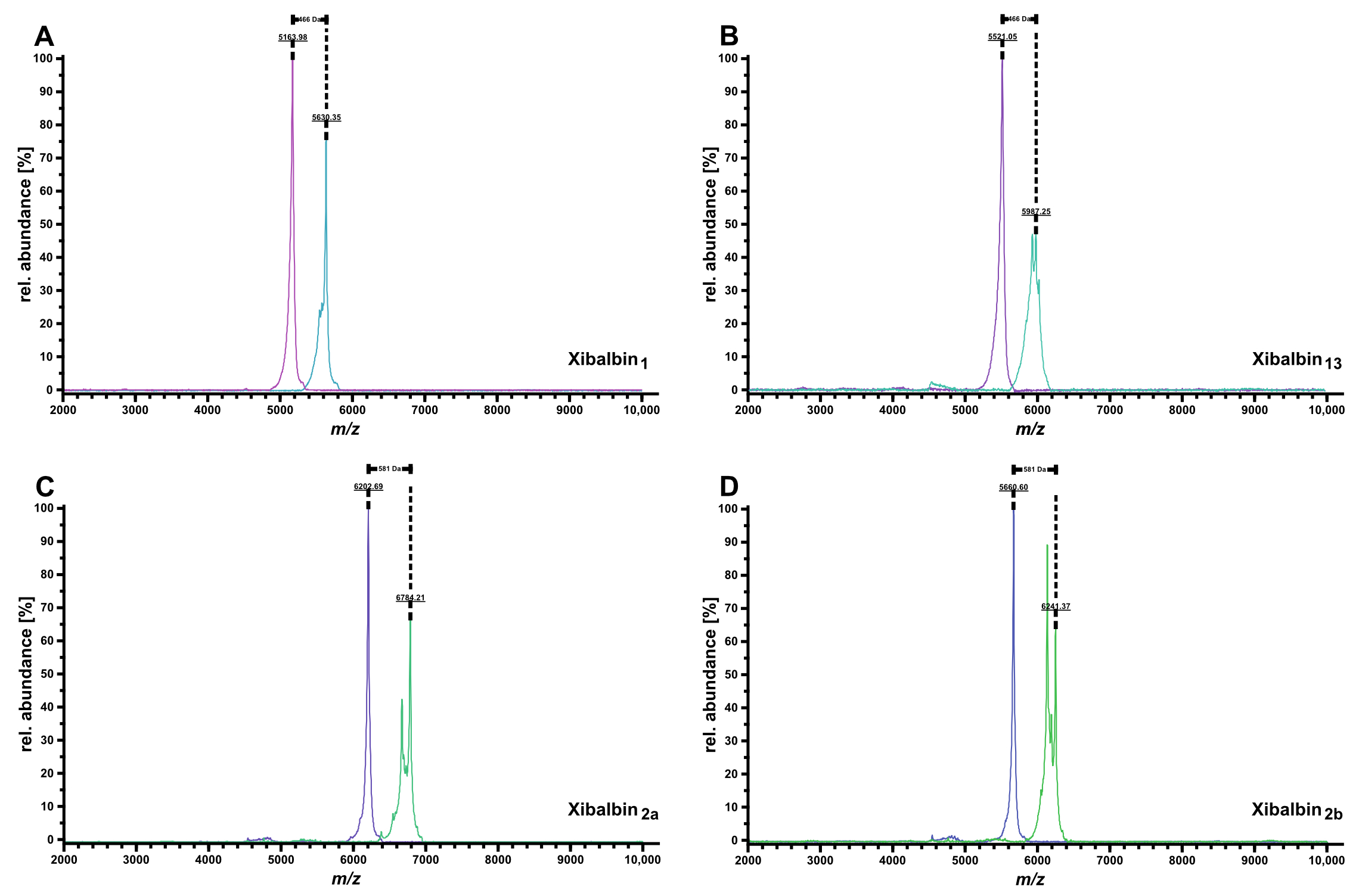

Supplement: Supplementary file 12 — Additional file 12: Figure S9. Dilsulfide bond matching of xibalbin variants by MALDI-ToF/ToF MS. [file 12915_2024_1955_MOESM12_ESM.jpg]
